# Advances in Noble Metal Electrocatalysts for Acidic Oxygen Evolution Reaction: Construction of Under‐Coordinated Active Sites

**DOI:** 10.1002/advs.202401652

**Published:** 2024-06-21

**Authors:** Huimin Wang, Zhenhua Yan, Fangyi Cheng, Jun Chen

**Affiliations:** ^1^ Key Laboratory of Advanced Energy Materials Chemistry (Ministry of Education), State Key Laboratory of Advanced Chemical Power Sources, College of Chemistry Nankai University Tianjin 300071 China

**Keywords:** electrocatalyst, noble metal, oxygen evolution reaction, PEMWE, under‐coordinated site

## Abstract

Renewable energy‐driven proton exchange membrane water electrolyzer (PEMWE) attracts widespread attention as a zero‐emission and sustainable technology. Oxygen evolution reaction (OER) catalysts with sluggish OER kinetics and rapid deactivation are major obstacles to the widespread commercialization of PEMWE. To date, although various advanced electrocatalysts have been reported to enhance acidic OER performance, Ru/Ir‐based nanomaterials remain the most promising catalysts for PEMWE applications. Therefore, there is an urgent need to develop efficient, stable, and cost‐effective Ru/Ir catalysts. Since the structure‐performance relationship is one of the most important tools for studying the reaction mechanism and constructing the optimal catalytic system. In this review, the recent research progress from the construction of unsaturated sites to gain a deeper understanding of the reaction and deactivation mechanism of catalysts is summarized. First, a general understanding of OER reaction mechanism, catalyst dissolution mechanism, and active site structure is provided. Then, advances in the design and synthesis of advanced acidic OER catalysts are reviewed in terms of the classification of unsaturated active site design, i.e., alloy, core‐shell, single‐atom, and framework structures. Finally, challenges and perspectives are presented for the future development of OER catalysts and renewable energy technologies for hydrogen production.

## Graphical abstract

Highly active, cost‐effective, and durable oxygen evolution reaction catalysts are indispensable for promoting the practical application of proton exchange membrane water electrolyzer. Understanding the structure‐activity relationship of catalysts is an important mean to study reaction mechanism and construct optimal catalytic system. In the review, the recent advanced catalysts are summarized based on the construction of unsaturated coordination sites to provide reference and ideas for the rational design of catalysts.

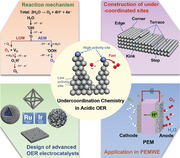

## 1 Introduction

As a clean secondary energy source, hydrogen (H_2_) energy has the advantages of large energy density, no pollution, and no carbon emissions. It can be used as an efficient, clean, and sustainable source to solve the energy crisis and build a clean, low‐carbon, safe, and efficient modern energy system.^[^ ^1^ ^]^ Compared with fossil energy and industrial tail gas reforming H_2_ production, electro‐chemical water splitting has become one of the most promising and effective ways to convert hydrogen energy, because of its high electrolysis efficiency, high hydrogen purity, and no pollution.^[^ ^2^ ^]^ In particular, the development of electrochemical conversion processes coupled with renewable energy (such as solar energy and wind), using water as raw materials to convert hydrogen energy, can help large‐scale renewable energy consumption and realize the power grid large‐scale peak shaving and cross‐season and cross‐regional energy storage.^[^ ^3^ ^]^

Recently, the technology of electro‐chemical water splitting mainly includes alkaline water electrolyzer (AWE), proton exchange membrane water electrolyzer (PEMWE), and solid oxide water electrolyzer (SOE).^[^ ^4^ ^]^ The SOE requires very high temperatures to transport carriers (i.e., O_2_ or H^+^) through the ceramic membrane electrolyte, whereas the other two operate significantly lower using aqueous electrolysis.^[^ ^5^ ^]^ Low‐temperature water electrolyzer technology is the greenest means of H_2_ preparation.^[^ ^6^ ^]^ Among them, AWE is more mature and has lower production costs, while PEMWE has a simple process and high energy efficiency.^[^ ^7^ ^]^ What's more, an intrinsic advantage of acidic electrolytes over alkaline electrolytes is that the conductivity of hydrated hydrogen ions (350 S cm^2^ mol^−1^) is significantly higher than that of hydroxide ions (198 S cm^2^ mol^−1^).^[^ ^8^ ^]^ Compared with commercially mature AWE, PEMWE based on acid electrolyte has many advantages such as higher current density, higher H_2_ purity, lower resistance loss, and more compact structure design, which is more suitable for practical applications.^[^ ^8^, ^9^ ^]^ In addition, the low gas permeability of the PEM helps to avoid gas cross‐penetration of hydrogen and oxygen, which can ensure a larger load range and safer operation of the acid electrolyzer.^[^ ^8^, ^9^ ^]^ PEMWE do not require tight control of pressure on both sides of the membrane, and have the advantages of fast start‐stop and fast power regulation response, which is suitable for the input of fluctuations in renewable energy generation.^[^ ^8e^ ^]^ In this context, PEMWEs are more attractive and desirable in future industrial applications, so acid electrolyzed water deserves more attention and efforts.^[^ ^10^ ^]^

Electrochemical water splitting consists of two half‐reactions: hydrogen evolution (HER) on the cathode side and oxygen evolution reaction (OER) on the anode side.^[^ ^1^, ^2^ ^]^ Compared with HER, which is relatively simple and prone to occur, OER involves a 4‐electron transfer process with slow kinetics, high reaction overpotential, and low energy conversion efficiency.^[^ ^3^, ^8^, ^11^ ^]^ At the same time, the harsh conditions in the OER process, including strong acid environment and oxidation environment, will trigger the catalyst to dissolve, which seriously affects the catalytic activity and stability under long‐term operation.^[^ ^12^ ^]^ Therefore, it is urgent to develop OER electrocatalysts with high activity and durable stability to achieve efficient and stable water electrolysis.

For OER catalysts, research on precious metals, alloys, metal oxides, doped modified polyoxides, porous nanostructures, and other materials has been undertaken.^[^ ^13^ ^]^ After a lot of research, scholars have found that even non‐precious transition metal‐based materials exhibit excellent catalytic activity and stability in alkaline media, and only a few of them have proven to be promising alternatives.^[^ ^14^ ^]^ However, compared with the activity and stability of noble metal catalysts in acidic media, there is still a big gap.^[^ ^15^ ^]^ At present, for OER catalysts on the anode of PEMWEs, the most suitable highly active acid electrocatalysts are still Ru‐based and Ir‐based catalysts.^[^ ^12^, ^16^ ^]^ Ru‐based catalysts are much more active than Ir‐based catalysts.^[^ ^16^, ^17^ ^]^ However, under the overpotential applied to the anode, it is easy to form RuO_4_ dissolved in solution, which in turn leads to reduced catalyst stability. For Ir‐based catalysts, although they are more stable under acidic conditions, they are less active and more expensive.^[^ ^16a^ ^]^ However, practical applications of PEMWE require the catalyst to operate stably for more than 50 000 h at 2.0 A cm^−2^, achieving a cost of US$2 per kg of H_2_. Thus, catalyst activity, stability, and high Ru/Ir loading (greater than 2.0 mg cm^−2^) are the main limiting factors for the large‐scale development of PEMWEs, and catalyst design requires a combination of activity, stability, and cost.^[^ ^7^, ^18^ ^]^ To overcome slow kinetics, reduce the energy consumption of electrolyzed water, and achieve the long‐term stability required for industrial applications, the intrinsic active species and material dissolution mechanism of the catalyst need to be clarified.

**Figure 1:**
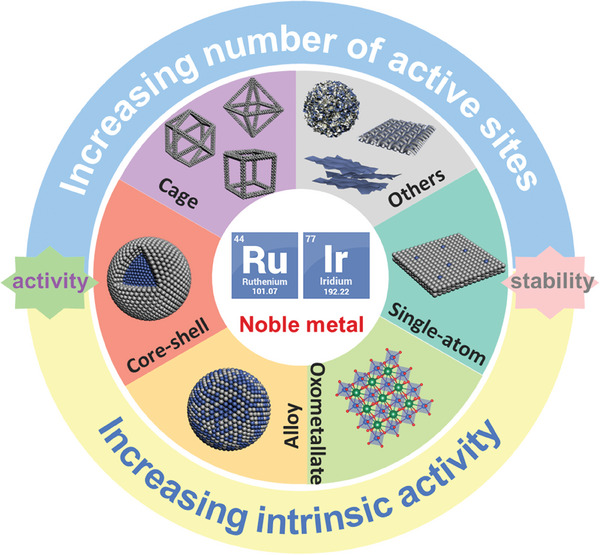
Classification of noble metal OER electrocatalysts in acid: construction of under‐coordinated active sites.

Given that understanding the structure‐activity relationship of metal catalysts is one of the important means to study the reaction mechanism and construct the optimal catalytic system. Here, we summarize the design of the nanostructure and structure‐activity relationship of acid OER catalysts. Unlike previous review articles mostly categorized pure metals and oxides, this review focuses on structural design. The comprehensive review of the construction of unsaturated sites for acid OER electrocatalysts can help researchers understand the structure‐activity relationship between structure and performance, and provide reference and ideas for the rational design of catalysts. First, the OER reaction mechanism and reaction‐dissolution mechanisms of catalysts are discussed, which can provide insight into OER and guidance for creating efficient and stable catalysts. Subsequently, the research progress of acidic OER catalysts was highlighted from the aspects of knowledge about electrocatalytic active sites, construction of under‐coordinated active sites, structure‐performance relationship (**Figure** **1**). Finally, the challenges of acidic OER development are proposed, and the design of more efficient OER electrocatalysts is prospected for future energy conversion and storage devices.

## 2 General Understanding about Acidic OER

### 2.1 Reaction Mechanism in Acidic Media

**Figure 2:**
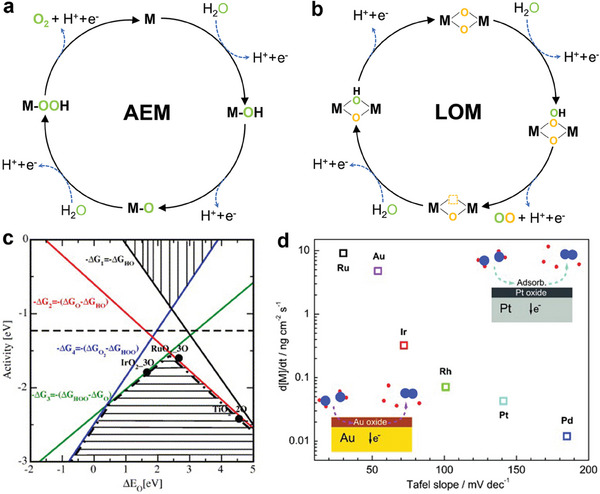
Illustration of the competition between a) the adsorbate evolution mechanism (AEM) and b) lattice‐oxygen participation mechanism (LOM). c) OER activity as a function of oxygen binding energy. Reproduced with permission.^[^ ^22^ ^]^ Copyright 2007, Elsevier Inc. d) Correlation between stability and the Tafel slope for the OER for the noble metals.^[^ ^16a^ ^]^

The construction of OER catalysts with excellent activity and stability must be carried out on the basis of understanding the catalytic reaction mechanism.^[^ ^8^, ^19^ ^]^ According to the difference of O‐O bond formation step, the reaction mechanism of acid OER can be divided into adsorbate evolution mechanism (AEM) and lattice oxygen evolution mechanism (LOM) (**Figure** **2**a,b).^[^ ^20^ ^]^ AEM considered that the oxygen produced comes from water in the electrolyte, while in LOM it comes partly from lattice oxygen in the catalyst.^[^ ^21^ ^]^

AEM involves a variety of oxygen reaction intermediates, such as ^*^OH, ^*^O, and ^*^OOH. As shown in Figure 2a, first, the adsorbed water oxidizes at the metal site to form *OH, and then deprotonates to form *O intermediates. Then, another water molecule nucleophilically attacks ^*^O, producing ^*^OOH intermediates. Finally, ^*^OOH intermediates are deprotonated to release O_2_ with regeneration of metal active sites. The binding energy of the intermediates is linearly related and follows the proportional relationship ΔG_*OOH_ = ΔG_*OH_ + 3.2 eV (Figure 2c).^[^ ^22^ ^]^ The binding energy of each intermediate cannot be adjusted independently, so a high overpotential (*η*) is still required to drive the reaction, and the minimum theoretical overpotential is ≈0.37 V.^[^ ^23^ ^]^

(1)

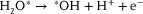



(2)

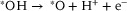



(3)

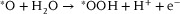



(4)

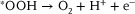




In the LOM mechanism, active oxygen in the lattice overflows from the lattice and interacts with the adsorbed oxygen (*O) to participate in the OER cycle. A typical LOM catalytic cycle is shown in Figure 2b, first, similar to the first two steps of AEM, the adsorbed H_2_O molecules are deprotonated twice in a row at the catalytic site to form O* intermediates. Next, O* coupled with lattice oxygen to form *OO intermediates. Subsequently, O_2_ is released and oxygen vacancies (V_O_) are formed.^[^ ^20^, ^24^ ^]^ Finally, the formed oxygen vacancies are regenerated by deprotonation of H_2_O molecules. The oxygen atoms in the water molecule fill the void of oxygen and undergo a process of deprotonation to form a new active site.

(5)

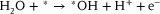



(6)

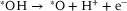



(7)

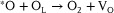



(8)

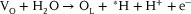



(9)

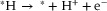




The LOM mechanism eliminates the collaborative proton electron transfer step that occurs in traditional AEM. It produces oxygen through direct coupling of O‐O bonds and does not involve the formation of *OOH, successfully breaking the scaling relationship in AEM.^[^ ^25^ ^]^ Moreover, the O‐O binding step in the LOM pathway is very easy, whereas this step is usually the rate‐determining step in AEM. Therefore, LOM‐compliant catalysts theoretically have higher OER activity than AEM‐compliant catalysts.^[^ ^20^, ^26^ ^]^ However, the formation of oxygen vacancies due to the LOM pathway accelerates the dissolution of metals, which triggers the collapse of the catalyst structure and thus leads to the rapid deactivation of the catalyst.^[^ ^20^, ^24^ ^]^ Therefore, catalysts following the LOM pathway tend to be relatively unstable.

Currently, it is believed that Ir‐based materials mainly follow the AEM mechanism with higher stability but less activity than Ru‐based materials (Figure 2d).^[^ ^16^, ^27^ ^]^ The unique LOM of Ru‐based oxides provides a significant breakthrough in activity, but the involvement of lattice oxygen leads to the formation of soluble high‐valent oxygen vacancy intermediates (^*^V_o_‐RuO_4_^2−^), which triggers catalyst structural collapse and dissolution, leading to a drastic decrease in catalytic activity and difficulty in maintaining stability.^[^ ^28^ ^]^ Under acidic conditions, inhibiting the LOM pathway at the expense of activity to improve the stability of Ru‐based electrocatalysts is currently the most common solution.

### 2.2 Understanding of Reaction‐Dissolution Mechanisms

With the development of in situ/operando characterization techniques and theoretical calculations, a large number of studies have been driven to unravel the reaction‐dissolution mechanism in dynamic electrocatalytic processes.^[^ ^28^, ^29^ ^]^ However, the complexity of the catalyst‐electrolyte interface leads to a stability‐activity mechanism in acidic media that is still not uniformly defined. A systematic study of the activity and stability of polycrystalline noble metals during OER oxidation has been carried out by Mayrhofer et al.^[^ ^16a^ ^]^ It is noteworthy that all metals exhibit both transient and steady‐state dissolution. Frequent voltage perturbations lead to the occurrence of transient dissolution during oxide formation/reduction.^[^ ^16a^ ^]^ In contrast, steady‐state dissolution depends on OER mechanism on each surface.

For catalysts that follow the AEM reaction mechanism, changes in the oxidation state can lead to slight transition metal dissolution during continuous operation.^[^ ^30^ ^]^ For example, there are at least three different dissolution mechanisms for the dissolution of Ir‐based materials during OER, including direct dissolution of Ir metals, the pathway through the Ir^V^‐Ir^III^ transition, and the formation of IrO_3_ at high potentials.^[^ ^31^ ^]^ The latter two of these mechanisms are closely related to the OER reaction mechanism. Differently, oxygen vacancies created during the LOM process will be replenished by water molecules or bulk oxygen atoms, while the resulting undercoordinated metal sites are more readily dissolved. The dissolution is more drastic in the more reactive polycrystalline RuO_2_ due to the drastic structural perturbation and weakening of metal‐oxygen bonds. There are two main reasons for the poor stability of Ru‐based catalysts at acidic OER, the first one is related to the oxidative release of lattice oxygen, which may lead to excessive oxidation of Ru to soluble RuO_4_ at the OER potential.^[^ ^12^, ^32^ ^]^ The second one is the direct demetallization of surface Ru, which can also lead to the collapse of the crystal structure of Ru‐based catalysts.^[^ ^27^, ^33^ ^]^

For a long time, an increase in catalytic activity has been achieved at the expense of long‐term stability, failing to achieve a balance between stability and activity.^[^ ^34^ ^]^ The practical application of PEMWE requires catalysts with both activity and stability, especially stability, which plays an indispensable role in achieving large‐scale commercial application of PEMWE.^[^ ^8^ ^]^ Simultaneous stabilization of lattice oxygen and surface Ru in RuO_2_ catalysts under OER conditions may be a viable approach to enhance their stability in acids.^[^ ^12^, ^27^, ^32^, ^33^ ^]^ Wang et al. significantly extended the durability of catalysts under acidic OER conditions by doping Ni into RuO_2_ nanocrystals.^[^ ^35^ ^]^ Density Functional Theory (DFT) studies and Operational Oxygen Differential Electrochemical Mass Spectrometry (DEMS) analyses confirmed that the Ni dopant enhances the lattice stability of surface Ru and subsurface oxygen. Based on the idea of reverse doping catalyst design, Xing's team^[^ ^36^ ^]^ doped atomic‐level Ti into the IrO_x_/Ir surface to reduce the dissolution of Ir by inhibiting the over‐oxidation of Ir species. Therefore, catalysts should be designed with an understanding of the properties of the catalytic active sites.

## 3 A Brief Summary of Current Knowledge about Electrocatalytic Active Sites

**Figure 3:**
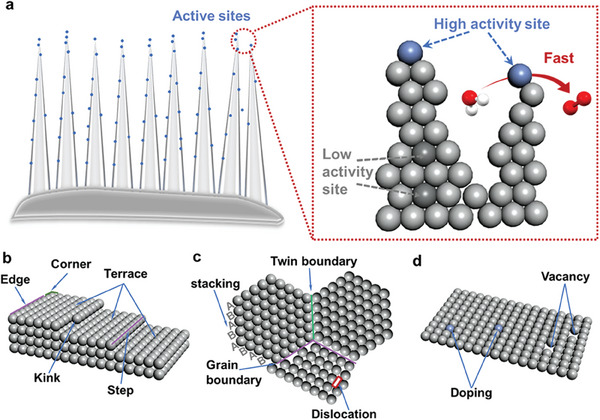
a) Schematic illustration of catalyst with high activity sites. b) Construction of under‐coordinated active sites, c) defect sites in materials, and d) doping and vacancies.

Catalytic active sites are certain surface sites that contribute to the activity of the catalyst. The surface atoms of active site are more active than other surface atoms and can catalyze specific chemical reactions (**Figure** **3**a).^[^ ^37^ ^]^ The characterization and identification of catalytic sites can help to understand the catalytic reaction mechanism, so as to better guide the design of electrocatalysts.^[^ ^29^, ^38^ ^]^ Initial research focused on identifying active sites by manipulating the size of catalysts to establish a relationship between the activity of these catalysts and their corresponding particle sizes.^[^ ^39^ ^]^ However, this indirect approach does not provide precise information about the active site. In recent years, a variety of in‐situ techniques for characterizing and monitoring catalyst surfaces and solid/liquid interfaces have emerged, such as attenuated total reflectance surface‐enhanced infrared absorption spectroscopy (ATR‐SEIRAS), surface enhanced Raman spectroscopy, scanning tunneling microscope (STM), in Situ X‐ray Absorption Spectroscopy (XAS), in Situ Ambient Pressure X‐ray Photoelectron Spectroscopy (APXPS), and so on.^[^ ^40^ ^]^ These advanced techniques enable a better understanding of the reaction mechanism by directly characterizing the active sites and adsorption intermediates on the catalyst surface. In addition, DFT calculations serve as a beneficial complement to experiments to help determine the active sites involved in various electrocatalytic reactions.^[^ ^41^ ^]^

With the development of nanocatalysis and the advancement of characterization technology, researchers have found that unsaturated coordination atoms on the surface of catalysts are often the active sites of catalysis, so the distribution and structure of atoms on the surface of the catalyst are adjusted by controlling the size, morphology, and crystal plane of nanocrystals to improve catalytic performance.^[^ ^37^, ^42^ ^]^ When the size of nanocrystals is reduced to atomic clusters and single atoms, their energy level structure and electronic structure will undergo fundamental changes.^[^ ^43^ ^]^ It is precisely because of this unique structural feature that single‐atom catalysts often exhibit different activity, selectivity, and stability than traditional nanocatalysts.^[^ ^13^, ^44^ ^]^ For electrocatalysts containing noble metals, their composition, crystal structure, and exposed crystal faces often determine the properties of their catalytic active sites.^[^ ^45^ ^]^ Correspondingly, optimizing the composition and crystal phase through processes such as alloying is a common strategy to improve activity, which can change the electronic structure and surface adsorption of the catalytic site, thereby promoting electrocatalysis and reducing the usage of noble metals required in the catalyst.^[^ ^45^, ^46^ ^]^ Besides, the surface adsorption capacity of the active site can be adjusted and the catalytic activity of the material can be improved by controlling the exposed surfaces and forming different atomic arrangements on different crystal faces.^[^ ^45^, ^47^ ^]^ For example, recent studies on Ru crystal faces have shown face‐dependent electrocatalytic activity toward OER, fcc‐Ru has more excellent OER performance compared to hcp‐Ru, and the (111) crystalline surface of Ru nanograins has better OER properties than the (100) crystalline surface.^[^ ^48^ ^]^

Atoms on solid surfaces are unsaturated with coordination, such as terraces, steps, kinks, edges, corner sites, surface defect sites, etc (Figure 3b). In addition, it has recently been shown that grain boundaries (GB) can be introduced into crystalline materials through stacked laminar dislocation motions or directional attachment during material growth,^[^ ^45d^ ^]^ and that the presence of GBs can enhance the catalytic activity of metals because not only compressive or tensile strains, but also novel types of strains can be flexibly introduced and controlled in such GB structures to optimize the catalytic performance, and the catalytic stability can be better preserved due to the reversibility of the GB structure (Figure 3c). Doping and vacancies contribute significantly to the catalytic activity of the materials (Figure 3d), such as, Rh doping combined surface oxygen vacancies can precisely regulate the lattice oxygen mediated mechanism‐oxygen vacancy site mechanism (LOM‐OVSM) via the Ru‐O‐Rh active sites of Rh‐RuO_2_, simultaneously boosting intrinsic activity and stability.^[^ ^49^ ^]^

The coordination number of atoms on the material surface is directly related to the adsorption energy, and the unsaturated atoms with low coordination number are more thermodynamically unstable and active in adsorbing different reaction intermediates than coordination number saturated atoms.^[^ ^37^ ^]^ Modulation of the local coordination structure of the catalytic site is of great interest and value because its OER behavior is closely related to the coordination unsaturated structure of the active center. Therefore, the active sites with unsaturated coordination sites can be maximally exposed on the catalyst surface by designing core‐shell structures, framework structures, atomic clusters, single atoms, etc., which can significantly improve the catalytic activity.

## 4 Advanced Electrocatalysts for Acidic OER

At present, the research of OER catalysts in acid electrolytes is still mainly focused on noble metals and their alloys, even if it has been found that non‐noble transition metal matrix materials have excellent catalytic activity and stability in alkaline media,^[^ ^14^ ^]^ the activity and stability in acidic media are still quite poor than noble metal catalysts.^[^ ^15^ ^]^ In addition, Ru and Ir‐based catalysts have excellent activity and applicable stability for OER in acidic media, compared to other platinum group metals such as Rh, Pd, and Pt.^[^ ^12^, ^16^ ^]^ Therefore, this review will focus on Ru and Ir‐based catalysts and the mechanism of their improved performance (Figure 1). In order to minimize the consumption of precious metals, the amount of precious metal catalysts used without sacrificing performance is minimized, so as to maximize their mass activity, while paying attention to the improvement of stability to achieve a balance between activity and stability.

There are two strategies to increase the activity of an electrocatalyst system: increasing the number of active sites on the electrode, such as increasing the loading or improving the catalyst structure to expose more active sites; Boosts the intrinsic activity of each active site.^[^ ^3b^ ^]^ These strategies are not mutually exclusive and ideally can be adjusted simultaneously to maximize activity. An increase in intrinsic activity leads directly to an increase in electrode activity, thus alleviating the transport problems associated with high catalyst loadings. And increased intrinsic activity reduces catalyst loading and lowers costs. The OER activity and stability of Ru‐ and Ir‐based catalysts are closely related to their intrinsic structures, i.e., the coordination structures of the Ru and Ir sites.^[^ ^13^, ^50^ ^]^ Surface unsaturated ligand atoms are used as catalytic sites, and the catalytic activity is improved by constructing surface unsaturated ligand atoms to increase the catalytic active sites. The main construction methods are alloy, core‐shell, framework structure, single atom, etc. In this section, acidic OER precious metal electrocatalysts are classified structurally to establish the constitutive relationship between structure and performance and provide ideas for the rational design of catalysts.

### 4.1 Alloys

Transition metal alloying of electrocatalysts serves as a well‐established protocol for modulating electrochemical properties at the atomic scale.^[^ ^51^ ^]^ In particular, the incorporation of transition metals can (1) change the surrounding electronic environment (e.g., *d*‐band centers); (2) increase the concentration of surface active species (e.g., surface OH groups); and (3) optimize the geometrical configuration of the active sites (e.g., atomic spacing, coordination number, etc.). With the introduction of foreign metals, lattice strains occur at specific interfaces and the charge will be redistributed, which in turn affects the coordination environment of atoms and increases the number of unsaturated coordination sites, thereby improving catalytic performance. In addition, the introduction of non‐precious metals not only adjusts the geometry and electronic structure, but also significantly reduces the use of precious metals.

OER is very sensitive to additional metal composition and therefore surface chemistry can be optimized by alloying with other transition metals, achieving mismatches in coordination environments, lattice parameters, and changes in charge distribution. Ru‐based alloys including Co‐RuIr,^[^ ^46b^ ^]^ RuB_2_,^[^ ^52^ ^]^ FeCoNiIrRu HEA nanocrystals,^[^ ^53^ ^]^ RuMn,^[^ ^54^ ^]^ E‐Ru/Fe ONAs,^[^ ^55^ ^]^ RuCu NSs,^[^ ^56^ ^]^ Ir_x_Ru_1−x_O_2_,^[^ ^57^ ^]^ and Ru_1–δ_Cu_δ_O_2–δ_^[^ ^58^ ^]^ with tunable morphology and electronic structure include good catalytic activity. Great efforts have been made to develop more stable Ru‐based catalysts, e.g., the study of low‐index crystalline surfaces.^[^ ^48^ ^]^ However, Ru‐based alloys have not yet met the requirements for practical applications due to poor stability under acidic OER conditions. Ir‐based bimetallic/polymetallic alloys have attracted much attention as they are more stable than Ru‐based materials and reduce the cost by reducing the amount of iridium used. Combined with morphological engineering liking construction of core‐shell structures, nanocages, polyhedra, nanowires, etc., such as IrCo nanodendrites,^[^ ^59^ ^]^ IrW nanodendritic structure,^[^ ^60^ ^]^ IrCo alloy nanodendrites with petal‐like architecture,^[^ ^61^ ^]^ IrNi alloy nanoflowers,^[^ ^62^ ^]^ P‐IrCu_x_ NCs,^[^ ^63^ ^]^ trimetallic IrNiFe,^[^ ^64^ ^]^ IrM (M = Ni, Co, Fe) Bimetallic Nanoclusters,^[^ ^65^ ^]^ (Mn_0.8_Ir_0.2_)O_2_,^[^ ^66^ ^]^ RuCu NSs,^[^ ^56^ ^]^ and np‐Ir_70_Ni_15_Co_15_,^[^ ^67^ ^]^ the number of unsaturated coordination active sites is greatly increased. Furthermore, leaching of less inert metals (Fe, Co, Ni) during acidic OER processes results in the generation of Ru/Ir low‐coordinated active sites, which are susceptible to nucleophilic attack by water or hydroxyl species, thus favoring oxygen evolution kinetics.

**Figure 4:**
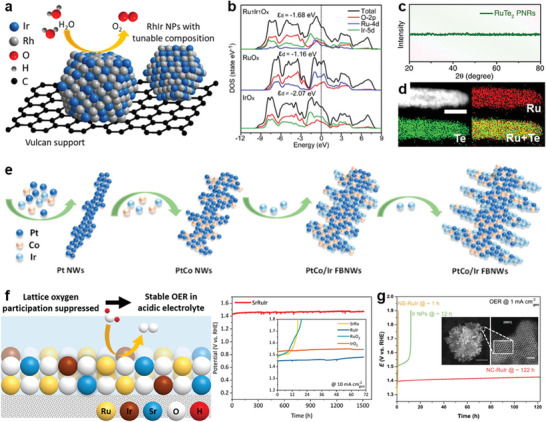
Noble alloy electrocatalysts. a) Schematic illustration of Rh_x_Ir_(100−x)_ NPs supported on Vulcan XC‐72R carbon. Reproduced with permission.^[^ ^68^ ^]^ Copyright 2016, American Chemical Society. b) The DOS curves for IrO_x_, RuO_x_, and Ru_1_Ir_1_O_x_. Reproduced with permission.^[^ ^69^ ^]^ Copyright 2021, Wiley‐VCH. c) XRD pattern and d) STEM‐ADF image and EDS elemental mappings of RuTe2 PNRs. Reproduced with permission.^[^ ^70^ ^]^ Copyright 2019, Springer Nature. e) Schematic shows the formation mechanism of PtCo/Ir FBNWs. Reproduced with permission.^[^ ^71^ ^]^ Copyright 2019, American Chemical Society. f) Scheme of stable OER in acidic electrolyte by suppressing lattice oxygen participation. Reproduced with permission.^[^ ^45b^ ^]^ Copyright 2021, American Chemical Society. g) Chronopotentiometric curves under the OER current density of 1 mA cm^−2^_geo_. Reproduced with permission.^[^ ^46a^ ^]^ Copyright 2021, Springer Nature.

A representative study on Rh‐Ir alloy nanoparticles by Yu and co‐workers revealed that the alloying of small amounts of Rh with Ir produces a synergistic combination of ensemble and electronic effects, which reduces the binding energy difference between O and OOH intermediates, thereby accelerating the kinetics and increasing the OER performance (**Figure** **4**a).^[^ ^68^ ^]^ The OER activity was found to be volcanically correlated with Ir composition, with the best performance being achieved by alloying 22% Rh into Ir, as evidenced by a 48 mV decrease in overpotential at a current density of 10 mA cm^−2^. In addition, the stability was greatly improved, and there was no significant decrease in OER activity after 2000 cycles. Recently Sun et al. developed an intermetallic Ru_1_Ir_1_O_x_ through incorporation of Ru into IrO_x_ (Figure 4b).^[^ ^69^ ^]^ Enhanced adsorption of oxygen intermediates was achieved by modulating the *d*‐band center and electronic structure of Ir, which dramatically improved the OER performance. Among them, the stability is excellent, with 110 h of stable operation at 100 mA cm^−2^_geo_.

Apart from the effect on electronic structure, alloying induces strain and changes the coordination environment. For example, Huang and his co‐workers verified that the local distortion‐strain effect can enhance the electron transfer of Ru‐sites by constructing amorphous RuTe_2_ alloys.^[^ ^70^ ^]^ The XRD pattern, STEM‐ADF image, and EDS elemental mappings of the catalyst demonstrated the RuTe_2_ PNRs were successfully fabricated (Figure 4c,d). This work demonstrates that the RuO_x_H_y_ species formed by the combination of high‐density defects with oxygen atoms facilitates OER, requiring only a cell voltage of 1.52 V to achieve a current density of 10 mA cm^−2^. Guo et al. reported a new class of Pt‐rich PtCo/Ir‐rich IrCo trimetallic fishbone‐like nanowires (PtCo/Ir FBNWs) with a unique surface/interfacial structure inducing interfacial stresses, which facilitates the effective combination of *d*‐band pinning and offsetting, and ultimately the activation of the OER (Figure 4e).^[^ ^71^ ^]^

Ru has excellent activity but poor stability, and Ir is stable but not as active as Ru. Ru_0.5_Ir_0.5_O_2_ with unsaturated coordinated Ru in catalyst surface can form more Ru active sites with high oxidation states at lower applied voltages after Ir incorporation, and the surface oxidative charge concentrations are increased, attributes its enhanced catalytic activity. In addition, the local structure of Ru‐O‐Ir prevented the excessive oxidative dissolution of the active site, dominated its high stability.^[^ ^72^ ^]^ Similarly, Sargent and co‐authors developed Sr‐Ru‐Ir ternary oxide electrocatalysts and analyzed the increased stability by X‐ray absorption spectroscopy and ^18^O isotope‐labeled online mass spectroscopy, where the lattice oxygen reaction was inhibited by interactions in the Ru‐O‐Ir local structure. Whereas Sr and Ir together modulated the electronic structure of the active Ru site and optimized the binding energy of the OER reaction intermediates. As a result, high OER activity and stability were achieved, with an overpotential of 190 mV at 10 mA cm^−2^ and an overpotential below 225 mV after 1,500 h of operation (Figure 4f).^[^ ^45b^ ^]^ Solid‐solution Ru‐Ir nanosized‐coral (RuIr‐NC) with the highest intrinsic activity and stability was reported by Kitagawa et al. (Figure 4g).^[^ ^46a^ ^]^ The high‐performance results from the ability of the preferentially exposed facets to resist the formation of dissolvable metal oxides and to transform ephemeral Ru into a long‐lived catalyst.

**Figure 5:**
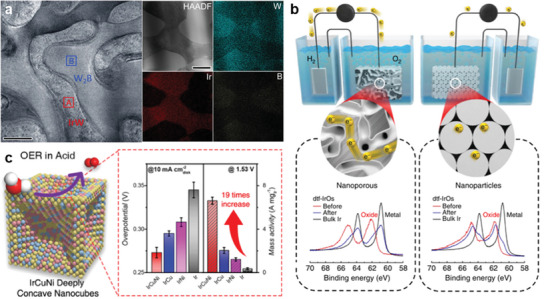
The design of noble alloy electrocatalysts. a) HAADF‐STEM image and the corresponding EDS elemental mapping of the W‐Ir‐B alloy catalyst. Reproduced with permission.^[^ ^13a^ ^]^ Copyright 2021, Springer Nature. b) Schematic illustrates the impact of multiple oxide‐oxide interfaces (present on dnp‐Ir_50_Os_50_ electrodes) on conductivity. Reproduced with permission.^[^ ^73^ ^]^ Copyright 2017, Springer Nature. c) Scheme of IrCuNi DCNCs with high activity toward OER. Reproduced with permission.^[^ ^74^ ^]^ Copyright 2021, American Chemical Society.

Notably, dealloying is an important part in the OER process, although de‐escaping‐induced surface reconstruction increases the complexity of the reaction sites, if properly utilized it will be a win‐win situation in terms of increased intrinsic activity and stability.^[^ ^54^ ^]^ The selective leaching of less inert components during dealloying gives the remaining noble metal a staggered structure, which provides a pathway for enhanced mass diffusion or electron transfer and more available internally active sites.^[^ ^75^ ^]^ Xiong and co‐workers reported a support‐stabilized catalyst with the activated IrW nanochannel (**Figure** **5**a).^[^ ^13a^ ^]^ The IrW support alters the charge distribution of the surface (IrO_2_)_n_ clusters and effectively prevents the agglomeration of surface Ir, achieving a win‐win strategy for ultra‐high OER activity and stability. A highly conductive nano‐porous architecture of an iridium oxide shell on a metallic iridium core was reported (Figure 5b),^[^ ^73^ ^]^ which was formed through the fast dealloying of osmium from an Ir_25_Os_75_ alloy. Quantification of the activity‐stability factor is proposed, and de‐alloyed nanoporous Ir_25_Os_75_ optimizes the stability and conductivity with an approximately 8‐fold increase in the activity‐stability factor. Another typical example is the preparation of IrCuNi deep concave nanocubes by selectively etching the facets of cubic nanoparticles, where the combination of high specific surface area, low coordination atoms on the stepped surface, and alloying effects result in excellent OER properties (Figure 5c).^[^ ^74^ ^]^

### 4.2 Core‐Shell

Compared to alloy structures, core‐shell structured catalysts can take advantage of their special lattice strain and ligand effects to optimize the geometrical and electronic properties of the surface shell metal. Unlike the coordination‐saturated bulk atoms, the surface atoms of the core‐shell structure are all coordination‐unsaturated. For this class of catalysts, electronic interactions between the two metals can promote the generation of the active species IrO_x_, and since coordination effects and charge transfer between the components are usually located at the core‐shell interface, modulation of the thickness of the shell layer can have a significant effect on the bimetallic synergism. Lattice strains in core‐shell bimetallic catalysts arise from lattice mismatches between the metal core and shell layers. Such lattice strains can usually be maintained within a few atomic layers and have a significant impact on the electronic structure of the shell metal, thus substantially modulating the catalytic performance of the catalyst. In addition, core‐shell catalysts enable more active site exposure, ultimately reducing the amount of precious metals used.

The construction of core‐shell structures has received much attention as a well‐established means, e.g., cladding Ru with a layer of IrO_x_ skin,^[^ ^76^ ^]^ which can apply compressive stress to the Ru core, thereby modulating the overall charge behavior and enhancing the OER activity of the material. However, the short‐range effectiveness of the interfacial charge distribution and stress action induced by the core‐shell structure suggests that the construction of sub‐nanoscale skins with several atomic layers is essential. Zhang et al. constructed a RuO_2_ sub‐nanoscale skin enriched with oxygen vacancies to encapsulate the core‐shell structure of a Ru cluster.^[^ ^77^ ^]^ Various other metals such as Ag, Ni, Fe, and Cu can also be used to construct core‐shell OER catalysts, a 3D hierarchical assembly structure composed of ultrathin Ru shell and a Ru‐Ni alloy core,^[^ ^78^ ^]^ IrGa‐IrO_x_,^[^ ^79^ ^]^ Ir‐Ag Nanotubes,^[^ ^80^ ^]^ IrRu@Te,^[^ ^81^ ^]^ α‐Ni(OH)_2_@Ir,^[^ ^82^ ^]^ Ir/Fe_4_N,^[^ ^83^ ^]^ Ir_x_Cu,^[^ ^84^ ^]^ IrNi@IrO_x_,^[^ ^85^ ^]^ Ru@Ir‐O,^[^ ^76b^ ^]^ and so on.

**Figure 6:**
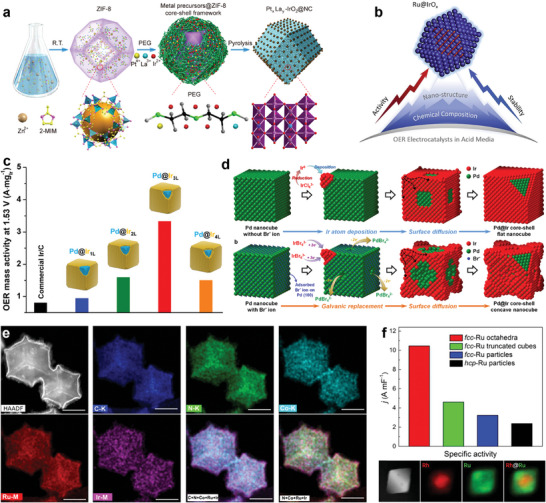
Noble core‐shell electrocatalysts, exposing more under‐coordinated active sites. a) Schematic illustration of employing ZIF‐8 as templates for synthesis of Pt_x_La_y_‐IrO_2_@NC. Reproduced with permission.^[^ ^86^ ^]^ Copyright 2020, Elsevier Ltd. b) Schematic of charge‐redistribution‐enhanced nanocrystalline Ru@IrO_x_ electrocatalysts for OER. Reproduced with permission.^[^ ^76a^ ^]^ Copyright 2019, Elsevier Ltd. c) Scheme of Pd@Ir_nL_ (*n* = 1–4) core‐shell Nanocubes for Highly efficient OER. Reproduced with permission.^[^ ^87^ ^]^ Copyright 2019, American Chemical Society. d) Schematic synthesis of Pd@Ir f‐nc and Pd@Ir c‐nc. Reproduced with permission.^[^ ^88^ ^]^ Copyright 2021, Elsevier Ltd. e) Elemental maps of C (K line), N (K line), Co (K line), Ru (M line), Ir (M line), and their overlay (C + N + Co + Ru + Ir and N + Co + Ru + Ir) of RuIr@CoNC catalysts. Reproduced with permission.^[^ ^89^ ^]^ Copyright 2021, American Chemical Society. f) HAADF‐STEM image and EDX mapping (Rh/red, Ru/green) of an individual octahedron. Reproduced with permission.^[^ ^48^ ^]^ Copyright 2019, American Chemical Society.

Core‐shell structure with exposing more under‐coordinated active sites on the catalyst surface greatly enhanced catalytic activity. An ultra‐small Pt, La co‐doped IrO_2_ nanoparticles highly dispersed on N‐doped carbon (NC) was designed.^[^ ^86^ ^]^ The structure achieved more active site exposure, and at the same time can rationally regulate the *d*‐energy band center and lower the energy barrier of the potential‐determining step (**Figure** **6**a). A rational design strategy for the fabrication of a heterostructured OER electrocatalyst (Ru@IrO_x_) was reported by Qiao and co‐author, demonstrated that an increase in the valence of the Ir shell and a decrease in the valence of the Ru core by inducing charge redistribution within the heterostructure, which activate synergistic electronic and structural interactions, leading to improved activity and stability (Figure 6b).^[^ ^76a^ ^]^ Since the catalytic reaction involves only a few layers of atoms on the surface, the atomic utilization of the precious metal atoms can reach up to 50% when the thickness of the precious metal cover is reduced to less than two atomic layers. A precise control over the thickness of the Ir shell from one to several atomic layers was reported by Xia's group (Figure 6c).^[^ ^87^ ^]^ The formation of a more stable phase by the Pd core during electrolysis, along with shell Pd‐Ir intermixing and ligand or strain effects, resulted in optimal activity and OER durability of the Pd@Ir_3L_ nanocubes. To investigate the surface structure‐electrochemical performance relationship, Lee et al. prepared Pd@Ir planar and concave nanocube models, one with a flat surface surrounded by (100) facets and the other with a concave surface containing numerous high‐index planes (Figure 6d).^[^ ^88^ ^]^ Similarly, Liu and co‐authors constructed surface atomic step‐controllable RuIr‐rich nanocrystals uniformly dispersed on the surface of MOF‐derived carbon support to simultaneously achieve improved electrocatalytic activity and stability (Figure 6e).^[^ ^89^ ^]^ The improved performance was attributed to the presence of a large number of atomic steps, i.e., unsaturated coordination sites, which maximally exposed the catalytically active sites.

The electrochemical properties of the material can be greatly influenced by controlling its crystal structure, exposing different crystalline surfaces, and controlling the ratio of exposure of different crystalline surfaces. However, due to the high adhesion and surface energy, Ru is generally hexagonal close‐packed (*hcp*) structure, and the synthesis of Ru nanoparticles with face‐centred cubic (*fcc*) and specific morphology (e.g., octahedral morphology) has been challenging. Xia et al. synthesized Ru nanoparticles with *fcc* structures by using 4.5‐nm Rh cubes as the seed for the growth and nucleation of Ru atoms, and the nanoparticles with mainly exposed (111) crystal faces (Figure 6f).^[^ ^48^ ^]^ And through a careful study of the OER properties, it is found that *fcc*‐Ru has better OER properties compared to hcp‐Ru, and Ru(111) facets have better OER properties than the (111) facets. The crystal surface modulation strategy can expose highly catalytically active crystal surfaces as well as increase the number of unsaturated coordination active sites on the catalyst surface, lowering the reaction energy barrier and increasing the catalytic reaction rate. It provides a research idea for the development of high‐performance OER catalysts.

**Figure 7:**
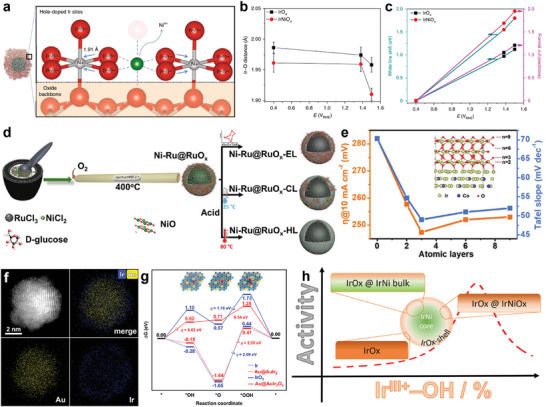
Noble core‐shell electrocatalysts, inducing generation of highly active oxides on the surface. a) Schematic of catalytic sites in IrNiO_x_ core–shell nanoparticles. b) Ir‐O bond distances at different applied potentials. c) Energy shift of the white line positions and formal Δ*d* in IrO_x_ and IrNiO_x_ nanoparticles at different electrode potentials compared with the corresponding sample at 0.4 V_RHE_. Reproduced with permission.^[^ ^90^ ^]^ Copyright 2018, Springer Nature. d) Synthesis procedure of the sample. Schematic of synthesis process of four different catalysts. Reproduced with permission.^[^ ^91^ ^]^ Copyright 2021, Wiley‐VCH. e) A graph showing the rule of overpotentials and Tafel plots of IrCo@IrO_x‐nL_ NDs varying along the atomic layers of IrO_x_. Reproduced with permission.^[^ ^59^ ^]^ Copyright 2019, Wiley‐VCH. f) HAADF‐STEM image and the corresponding EDX mapping of Au@AuIr_2_. g) Free energy diagram for OER on clean metallic species Ir, Au@AuIr_2_, oxidized species IrO_x_, and Au@AuIr_2_O_x_ at U = 1.23 V versus RHE. Reproduced with permission.^[^ ^13b^ ^]^ Copyright 2021, American Chemical Society. h) Scheme of the correlation between Ir^III+^‐ and OH‐ surface concentration of the oxide catalyst with OER activity. Reproduced with permission.^[^ ^92^ ^]^ Copyright 2019, American Chemical Society.

More importantly, the unsaturated coordination atoms on the surface of the core‐shell structure are susceptible to the formation of the reactive species IrO_x_ upon bimetallic electronic interactions. In particular, lattice vacancies are formed by the leaching of non‐precious metals in the electrocatalytic process, resulting in the formation of highly active amorphous surface layers of Ir oxides. Recently, Strasser et al. used using operando X‐ray absorption spectroscopy, resonant high‐energy X‐ray diffraction, and differential atomic pair correlation analysis to probe the effects of nickel leaching on the local geometric ligand environment and electronic metal states of oxygen‐coordinated iridium centers in IrNi@IrO_x_ core‐shell NPs under OER conditions (**Figure** **7**a).^[^ ^90^ ^]^ Nickel leaching during catalyst activation generates lattice vacancies, which in turn produce uniquely shortened Ir‐O metal ligand bonds and an unusually large number of *d*‐band holes in the iridium oxide shell (Figure 7b,c). Kim et al. generated unsaturated coordination atoms by leaching a certain amount of Ni from a Ru‐based core‐shell catalyst (Ni‐Ru@RuO_x_), which accelerate the LOM (Figure 7d).^[^ ^91^ ^]^ Unique acid‐base and direct‐coupled LOMs and AEM were elucidated under different surface defect engineering conditions. It was found by in situ electrochemical tests combined with DFT calculations that high‐index surfaces such as Ir (553) are thermodynamically favorable for the adsorption of oxygen atoms and the formation of oxides, and concave iridium surfaces with a large number of high‐index planes are susceptible to the formation of surface iridium oxides during the OER process, and therefore, their OER activity gradually increases.

Strain regulation is capable of tuning the surface chemistry of nanocatalysts to optimize catalytic performance. Atomic layers of IrO_x_ were constructed on the surface of IrCo nanodendrites with tunable Ir‐O bond length due to compressive strain effects (Figure 7e).^[^ ^59^ ^]^ It balances the adsorbate‐substrate interaction and facilitates the rate‐determining step of HOO* formation. At present, some researches have shown amorphous IrO_x_ showed higher OER activity and stability than rutile IrO_2_. Therefore, the construction of amorphous IrO_x_ has become the focus of research. Recently, Cao et al. reported novel core‐shell structure NPs with Au core and AuIr_2_ alloy shell (Au@AuIr_2_) (Figure 7f).^[^ ^13b^ ^]^ They demonstrated that the core‐shell structure exposing unsaturated coordination of Ir as well as the interaction between Au and Ir lead to the formation of partially oxidized surface, providing an equilibrium for the binding of different intermediates and achieving significantly enhanced OER activity and stability (Figure 7g). The intrinsic activity of Au@AuIr_2_ is significantly up to 4.6 times higher than the commercial Ir catalyst toward OER. However, the exact nature of the active sites for these reactions remains a matter,^[^ ^93^ ^]^ and all Ir^3+^, Ir^4+^, and Ir^5+^ have been considered as active sites.^[^ ^30^, ^94^ ^]^ Strasser et al. first found a close correlation between the composition of Ir^III+^ and OH^−^ surface concentrations on the catalyst surface, determined the experimental volcano relationship for Ir‐based OER electrocatalysts, and revealed universal OER activity predictors (Figure 7h).^[^ ^92^ ^]^

### 4.3 Nanoframes

Benefiting from the presence of a large number of unsaturated coordination sites such as steps, kinks, edges, corners, surface defect sites, etc. in the framework structure, which provides plenty of accessible active sites. Most of the atoms in the nanoframe catalyst can participate in the reaction, Thus, nanoframe structures can largely reduce the usage of noble metals. More importantly, the hollow structures with tunable components can optimize the adsorption of intermediates and desorption of products to improve the intrinsic activity of each active site.^[^ ^95^ ^]^ The open framework structure can be reconfigured into ultra‐small, surface hydroxylated active species during the OER process. For example, open‐framework iridate‐derived catalysts can generate crystalline active phases with very low iridium dissolution and ultra‐high stability.^[^ ^96^ ^]^ In principle, the framework structure is susceptible to torsional strain, and the adjustment of the coordination structure to produce low‐coordinated Ru/Ir atoms can essentially modulate the catalytic performance of the active site. More importantly, the twisted structure of the edge/face shared structure reduces the energy barrier of the rate‐limiting step.^[^ ^97^ ^]^ The design and synthesis of framework‐structured OER catalysts have now seen significant development and noteworthy achievements.^[^ ^98^ ^]^

**Figure 8:**
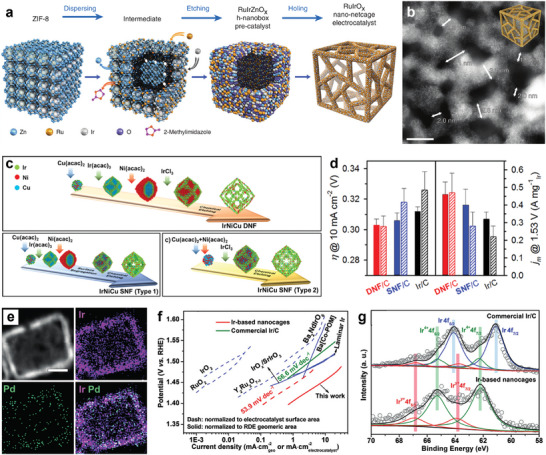
Noble frame structure electrocatalysts. a) Schematic illustration of the synthetic process for RuIrO_x_ nano‐netcage electrocatalyst. b) AC HAADF‐STEM and magnified images of RuIrO_x_ nano‐netcages. Scale bar: 5 nm. Reproduced with permission.^[^ ^99^ ^]^ Copyright 2019, Springer Nature. c) Schematic illustration of precursor‐type‐dependent formation of IrNiCu DNF and SNF structures. d) Bar graph showing overpotential (*η*) to drive 10 mA cm^−2^ and Ir mass activity (*j*_m_) at 1.53 V (versus RHE) of the catalysts. Patterned bar indicates the activity parameters after the durability test. Reproduced with permission.^[^ ^98^ ^]^ Copyright 2017, American Chemical Society. e) EDX mapping of Ir and Pd (scale bar: 5 nm). f) Tafel plot of the nanocages, commercial Ir/C and other representative electrocatalysts in acidic media. g) XPS spectra of Ir 4f recorded from Ir‐based nanocages and commercial Ir/C after 500 cycles of durability test (the value of *y* was greater than 4). Reproduced with permission.^[^ ^13c^ ^]^ Copyright 2019, Wiley‐VCH.

Metal‐organic frameworks (MOFs), which have the advantage of being structurally and chemically tunable, are assembled from metal ions/clusters with organic ligands and are considered as ideal templates for nanoframe structures. Li and co‐authors chose the most common MOF‐ZIF‐8 and adopted a dispersing‐etching‐holing strategy to obtain 3D RuIrO_x_ porous nano‐netcage by electrochemical in‐situ etching of ZIF‐8 with uniformly dispersed Ru/Ir active components (**Figure** **8**a).^[^ ^99^ ^]^ This structure improves the exposure of the active site and the 3D accessibility of the substrate molecule (Figure 8b), and the introduction of Ir effectively inhibits the over‐oxidation of Ru, thereby protecting the OER activity from decreasing. A more structurally robust Ir‐based multimetallic double‐layered nanoframe (DNF) structure was designed by park et al. A simple one‐step method was used to synthesize the core‐shell alloy@alloy structure by exploiting the kinetic differences between dual Ir precursors and dual transition metal (Ni and Cu) precursors, followed by selective etching to obtain the multimetallic IrNiCu DNF (Figure 8c).^[^ ^98^ ^]^ The IrNiCu DNF exhibits excellent OER activity and durability, which is attributed to the framework structure that increases the number of unsaturated coordination active sites while inhibiting particle growth and agglomeration as well as the in situ formation of a robust rutile IrO_2_ phase during prolonged operation (Figure 8d).

Single‐crystal Cu‐Ir polyhedral nanocages (NCs) not only significantly reduce the loading of Ir, but also form a unique NC structure that tunes the *d*‐band structure of Ir, thus significantly improving its OER catalytic activity.^[^ ^100^ ^]^ However, precise regulation of the thickness of nanoframes remains a major challenge, Zhu et al. successfully prepared an Ir_44_Pd_10_ cubic nanocage with well‐defined {100} facets and a thickness of ≈1.1 nm (Figure 8e).^[^ ^13c^ ^]^ The performance of the nanocage was considerably better than all OER catalysts reported in the literature (Figure 8f), with an overpotential of only 226 mV to reach 10 mA cm^−2^_geo_. Moreover, the catalyst showed 18.1‐fold and 26.2‐fold enhancement in mass (1.99 A mg^−1^_Ir_) and specific activity (3.93 mA cm^−2^_Ir_), respectively, as compared to Ir/C. The improved performance is attributed to the high utilization efficiency of Ir atoms in the ultra‐thin‐walled nanoframe and the formation of unsaturated sites in the open structure, which facilitates the electrochemical oxidation of Ir to the active form of IrO_x_ (Figure 8g). The group also reported a trimetallic cubic nanocage Pt_39_Ir_10_Pd_11_, which shows an OER mass activity of 0.20 A mg^−1^_Pt+Ir_ at 1.53 V. The highly open structure of the nanocage and the possible electronic coupling between Ir and Pt atoms in the lattice contributed to the significant mass activity.^[^ ^101^ ^]^

### 4.4 Single‐Atom

Single‐atom with unique electronic structures and unsaturated coordination environments can not only expose abundant active catalytic sites and higher atom usage, endowing single‐atom catalysts with superior catalytic performance.^[^ ^102^ ^]^ Moreover, it can provide an atomic‐scale perspective to understand the relationship between catalytic activity and structural properties.^[^ ^103^ ^]^ In particular, with the development of characterization techniques,^[^ ^104^ ^]^ characterization of catalysts at the atomic level has been achieved, and single‐atom catalysts have attracted a great deal of attention due to the design and optimization of ideal structures of catalysts on an atomic scale.^[^ ^105^ ^]^ Single‐atom OER catalysts hold the promise of perfect atom utilization, yet control of their activity and stability remains challenging. In recent years researchers have carried out a great deal of research around the design of single‐atom OER catalysts, aiming for a triple win in terms of activity, stability, and cost.^[^ ^13^, ^29^, ^45^, ^106^ ^]^

**Figure 9:**
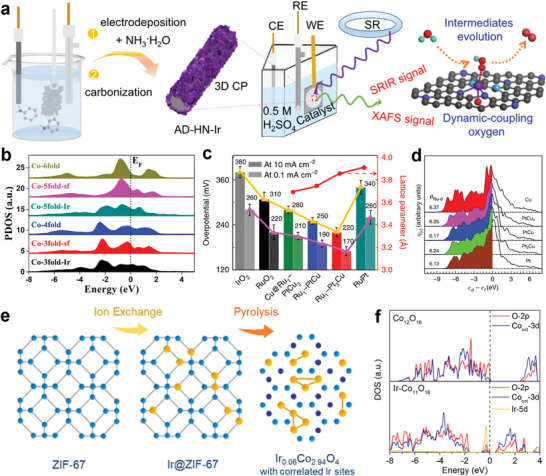
The design of noble single‐atom electrocatalysts. a) Scheme of the synthetic process. CE counter electrode, WE, working electrode, RE reference electrode, CP carbon paper, SR synchrotron radiation, C black, N_1_ royal blue, N_2_ indigo, O red, H green, Ir purple. Reproduced with permission.^[^ ^107^ ^]^ Copyright 2021, Springer Nature. b) PDOSs of Co‐3d bands from bulk lattice toward surface region. Reproduced with permission.^[^ ^108^ ^]^ Copyright 2020, American Chemical Society. c) Overpotential to reach 0.1 mA cm^−2^ and 10 mA cm^−2^ for the catalysts (left axis) and lattice parameter dependence on the composition of Pt/Cu (right axis, red line). Error bars show the s.d. evaluated from five independent measurements. d) PDOS of surface‐embedded Ru_1_ 4*d* with respect to the Fermi level, where *e*_Ru‐d_ is total 4*d* electrons of the Ru_1_ atom. Reproduced with permission.^[^ ^44^ ^]^ Copyright 2019, Springer Nature. e) Schematic illustration of the synthetic process for Ir_0.06_Co_2.94_O_4_ catalyst. f) Partial DOS of pure Co_12_O_16_ (upper) and Ir‐Co_11_O_16_ (lower). Reproduced with permission.^[^ ^109^ ^]^ Copyright 2021, American Chemical Society.

Single‐atom catalysts can be designed by precise modulation of the unsaturated coordination around the single atoms, size effects, and local electron density.^[^ ^110^ ^]^ However, when the metal particles are reduced to the single‐atom level, the specific surface area increases dramatically, leading to a sharp increase in the free energy of the metal surface.^[^ ^111^ ^]^ Agglomeration coupling is prone to form large clusters during preparation and reaction, which leads to catalyst deactivation, making the preparation of single‐atom catalysts a great obstacle and challenge. Currently, single‐atom catalyst synthesis strategies are classified into three main categories, including vacancy defect anchoring strategy, space‐limited domain strategy, and coordination design strategy.^[^ ^112^ ^]^ Wei and co‐authors reported a novel atomically dispersed hetero‐nitrogen‐configured Ir sites (AD‐HN‐Ir), and demonstrated that the Ir active site forms an oxygen atom in an O‐hetero‐Ir‐N_4_ structure by in‐situ synchrotron radiation infrared and X‐ray absorption spectroscopies (**Figure** **9**a).^[^ ^107^ ^]^ The structure acts as a more electrophilic active center, facilitates the generation of key *OOH intermediates, and inhibits peroxidation and solubilization of the active site, thus improving the activity and stability of acidic OER. Recently, Yin et al. introduced Ir single‐atoms into ultrathin NiCo_2_O_4_ porous nanosheets using co‐electrodeposition to produce Ir−NiCo_2_O_4_ NSs.^[^ ^108^ ^]^ Surface‐exposed less‐coordinated Ir single atoms were coupled with oxygen vacancies to obtain more active catalytic sites (Ir‐O_x_), with high electron exchange and transfer activity in the vicinity of the Ir‐O_x_ active sites. At the same time, the formation of Ir‐O_x_ active intermediates provides protection for the stabilization of the internal spinel structure under acidic conditions (Figure 9b).

Notably, single atom‐support interactions also have an important effect on catalytic performance, Wu's team successfully prepared Ru single‐atom catalysts using surface defect engineering techniques to capture and stabilize single atoms.^[^ ^44^ ^]^ A volcanic relationship between activity and PtCu alloy lattice constants was found by comparing the OER performance of a range of alloy‐supported Ru_1_ catalysts (Figure 9c). The compressive strain of the Pt_skin_ shell layer can alter the electronic structure of Ru_1_ and optimize the oxygen binding, which accelerate OER reaction kinetics and providing better resistance to peroxidation and dissolution (Figure 9d). Metal atoms dispersed on oxide supports form a large class of single‐atom catalysts.^[^ ^106^, ^113^ ^]^ High‐temperature pyrolysis of MOFs is another important method for obtaining single‐atom catalysts (Figure 9e).^[^ ^109^ ^]^ This work enables substitutional doping of noble metal Ir in transition metal oxide Co_3_O_4_ by ion‐exchange‐pyrolysis synthesis strategy using ZIF‐67 as a carrier. Moreover, most of the Ir sites show short‐range ordered structures. Ir doping creates a new energy band Ir 5*d*‐O 2*p* in the energy band gap, which would promote the charge transfer rate between the reaction intermediates and the catalyst surface (Figure 9f). Moreover, the ordered Ir sites can make the *d*‐band center of the active site lower, which can effectively regulate the adsorption strength of the intermediate and facilitate the catalytic activity. In addition, Ir doping leads to the downward shift of the valence band top of Co_oct_, which is less likely to lose valence electrons and be oxidized, and thus the stability is enhanced. Chorkendorff et al. developed an iridium‐tantalum oxide (Ir‐Ta‐O) catalyst based on stabilized Ta_2_O_5_ nanoclusters, and the OER performance study revealed that the special iridium coordination and reduced free energy of hydrolysis enhanced the performance.^[^ ^114^ ^]^

**Figure 10:**
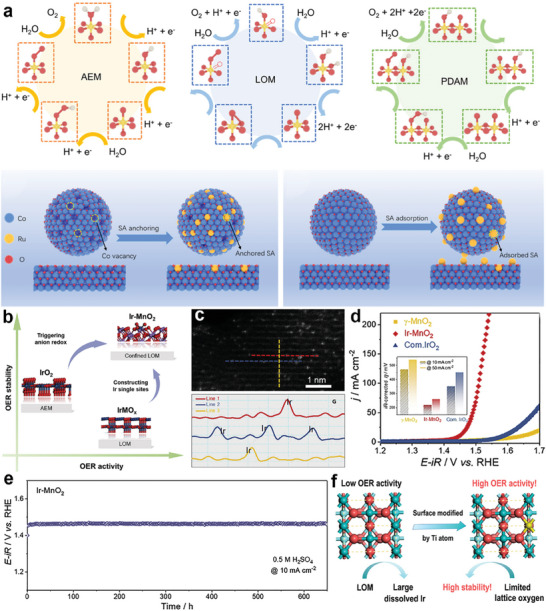
The OER mechanism of noble single‐atom electrocatalysts. a) Illustration of the catalytic mechanisms of atomically dispersed ruthenium sites.^[^ ^115^ ^]^ Copyright 2023, American Chemical Society. b) The design of an Ir single‐atom catalyst can trigger lattice oxygen oxidation locally while maintaining the stability of the bulk structure. c) The lines represent the line profiles for HAADF intensity analysis. d) Representative LSV curves of g‐MnO_2_, Ir‐MnO_2_, and commercial IrO_2_ in 0.5 m H_2_SO_4_. e) Chronopotentiometric response of Ir‐MnO_2_ for OER at 10 mA cm^−2^. Reproduced with permission.^[^ ^116^ ^]^ Copyright 2021, Elsevier Ltd. f) Illustration of the OER mechanism of IrO_x_/Ti.^[^ ^36a^ ^]^ Copyright 2023, Elsevier Ltd.

An in‐depth understanding of the oxygen precipitation mechanism at single atom sites can provide feedback to guide the optimization of local structures and the design of ideal catalysts. Recently, Peng et al. have precisely controlled the spatial position of Ru single atoms by utilizing metal defect anchors on Co_3_O_4_ supports.^[^ ^115^ ^]^ Especially, Ru atoms embedded in cationic vacancies reveal an optimized mechanism for activating proton donor‐acceptor function (PDAM), breaking the classical scaling relation of OER (**Figure** **10**a). This research found that spatial interactions were able to optimize the conformation of the intermediates as well as lower the energy barriers. Thus, fine structural design can transform the reaction pathway. Similarly, Ge et al. prepared a novel Ir single‐atom catalyst embedded in γ‐MnO_2_ with localized and isolated Ir‐O covalent bonds by carefully designing the coordination structure of Ir (Figure 10b,c).^[^ ^116^ ^]^ The structure enhances the Ir‐O bond covalency and locally triggers the lattice oxygen redox, thereby increasing the OER activity (Figure 10d). Moreover, the locally activated lattice oxygen oxidation does not cause any ontological structural evolution or damage during the OER process, and is stable for up to 650 h (Figure 10e). This study provides a novel strategy for triggering localized LOM to increase intrinsic activity and stability, which provides theoretical implications for the design of efficient and stable catalysts in the future. Another single‐atom OER catalyst construction idea is the inverse doping strategy, such as doping Ti atoms into IrO_x_/Ir matrix to form rich Ir‐O‐Ti motifs (Figure 10f).^[^ ^36a^ ^]^ The Ti sites provide electrons to weaken Ir‐O interactions through AEM, thus stimulating the activity of Ir sites. Besides, the restricted O‐O bond formation in the LOM and the expansion of the stabilization region of Ir species contribute to the enhanced stability. This work provides new insights into the construction of atomic‐level interfacial motifs in long‐lived efficient catalysts.

### 4.5 Other Structures

Apart from the above core‐shell, single‐atom, and framework structures, a large number of edges, corners, and other unsaturated coordinated sites are exposed in the structures such as ultrasmall nanoparticles, clusters, and ultrathin nanosheets. Surface modification to construct surface unsaturated sites by defect engineering, crystal surface modulation to improve active site exposure, doping, and supports to construct coordination‐unsaturated metal‐support interfacial sites, all of which are capable of constructing highly active coordination‐unsaturated catalytic sites. And coordinated unsaturated metal ions present in structures such as oxometallate can also serve as high‐performance catalytically active sites.^[^ ^117^ ^]^ In this chapter, the above structures will be reviewed to provide a reliable basis for the preparation of new catalysts in the future.

#### 4.5.1 Typical Oxometallate‐Type Structure

**Figure 11:**
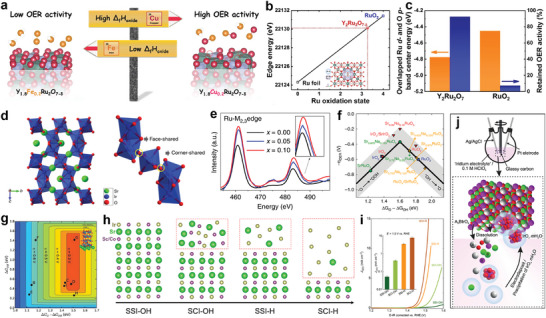
The typical oxometallate‐type structure. a) Scheme of A‐site substituent in yttrium ruthenium pyrochlores Y_1.8_M_0.2_Ru_2_O_7−δ_ (M = Cu, Co, Ni, Fe, Y) controlling the concentration of surface oxygen vacancies (V_O_) with OER activity. Reproduced with permission.^[^ ^118^ ^]^ Copyright 2020, American Chemical Society. b) Relationship between Ru K‐edge energy and oxidation state for Y_2_Ru_2_O_7‐δ_ and the two reference materials. c) Comparison of overlapped band center energy of Ru 4d and O 2p orbital, and activity loss of current density at 1.50 V for Y_2_Ru_2_O_7_ and RuO_2_, respectively. Reproduced with permission.^[^ ^119^ ^]^ Copyright 2017, American Chemical Society. d) Crystal structure of 6H‐SrIrO_3_ and a local connection pattern of IrO_6_ octahedra. Reproduced with permission.^[^ ^120^ ^]^ Copyright 2018, Springer Nature. e) Ru M_2,3_‐edge XANES spectra for SrRuO_3_ (black), Sr_0.95_Na_0.05_RuO_3_ (blue) and Sr_0.90_Na_0.10_RuO_3_ (red). f) OER Volcano‐type activity plot. Reproduced with permission.^[^ ^121^ ^]^ Copyright 2019, Springer Nature. g) Theoretical overpotential (*η*) volcano plot with O^*^ and OH^*^ binding energies as descriptors. Reproduced with permission.^[^ ^122^ ^]^ Copyright 2016, American Association for the Advancement of Science. h) Schematic of the surface status in all four samples. i) BET‐normalized activities from SSI‐OH, SCI‐OH, SSI‐H, and SCI‐H. The inset shows the BET‐normalized OER currents at 1.5 V versus RHE. Reproduced with permission.^[^ ^123^ ^]^ Copyright 2021, American Association for the Advancement of Science. j) Experimental set‐ups and the proposed OER catalytic mechanism involving the “dissolution‐electrodeposition” of iridium species. Reproduced with permission.^[^ ^124^ ^]^ Copyright 2019, Wiley‐VCH.

Pyrochlore materials with the general formula A_2_B_2_O_7−δ_ (A is typically a rare‐earth or alkaline‐earth metal, B is a transition metal) have emerged recently as promising alternatives to binary RuO_x_ and IrO_x_ oxides owing to their lower noble metal content and high activity in acidic OER.^[^ ^106^, ^119^, ^125^ ^]^ Müller et al. selected model pyrochlores with the nominal composition Y_1.8_M_0.2_Ru_2_O_7−δ_ (M = Cu, Co, Ni, Fe, Y) for OER catalysis^[^ ^118^ ^]^ and demonstrated that the A‐site substituent controlled the concentration of surface oxygen vacancies (V_O_). whereby an increased concentration of V_O_ sites correlates with a superior OER activity (**Figure** **11**a). The weaker the A‐site substituent M‐O bond strength, the higher V_O_ surface density, leading to better OER properties. Another work indicated Y_2_Ru_2_O_7‐δ_ catalyst had a low valence state of Ru (Figure 11b) that favored the high OER activity and lower central energy of the overlapping energy bands of the Ru 4*d* and O 2*p* orbitals (Figure 11c), resulting in a more stable Ru‐O bond with higher stability.^[^ ^119^ ^]^ Similarly, great efforts have been made to enhance the activity and stability of iridates, investigating the effect of the presence of metal bonds on the local structure of the material and the modulation of the conductivity and catalytic activity sites.^[^ ^120^, ^126^ ^]^ Zou and co‐authors^[^ ^120^ ^]^ presented an unusual perovskite oxide catalyst 6H‐phase SrIrO_3_ (6H‐SrIrO_3_) with face‐sharing IrO_6_ octahedral dimers (Figure 11d). The structure of the face‐sharing IrO_6_ octahedral dimers weakens surface Ir‐O binding and facilitates the OER potential determination step.

The high activity of perovskite usually comes at the expense of structural stability, with active site desolvation and surface remodeling during acidic oxygen precipitation being more prominent. Thus, Rojas et al. modified SrRuO_3_ by Na doping,^[^ ^121^ ^]^ which increased the oxidation state of Ru with positively displaced the O *p*‐band and Ru *d*‐band centers, thereby weakening the Ru‐adsorbate bond. Along with a slightly higher oxidation state of the Ru center, higher solvation potential, lower surface energy, and less distorted RuO_6_ octahedra (Figure 11e). Thus, doping achieved high OER activity and durability simultaneously (Figure 11f). Zou's group reports an Ir‐doped activated SrTiO_3_ making inert metal Ti available as an active site.^[^ ^106e^ ^]^ Ir doping can effectively modulate the electronic structure of SrTiO_3_, producing Ir‐O hybrid electronic states spanning the Fermi energy level. SrTiO_3_, as a robust host material, is able to stabilize the catalytic OER in acidic conditions while maintaining its own crystal structure.

Metal leaching in perovskites affects stability, but the characteristic surface reconstruction allows the catalyst to exhibit superior OER activity.^[^ ^125^, ^127^ ^]^ Jaramillo et al.^[^ ^122^, ^128^ ^]^ reported an IrO_x_/SrIrO_3_ catalyst formed by strontium leaching from surface layers of SrIrO_3_ thin films during electrochemical testing (Figure 11g). Highly active IrO_x_ surface layer formed in situ, resulting in the catalyst with excellent stability and high intrinsic activity. Furthermore, leaching of the perovskite A/B sites results in entirely different catalyst properties. Xu and co‐authors^[^ ^123^ ^]^ investigated the occurrence and process of surface reconstruction using SrSc_0.5_Ir_0.5_O_3_ and SrCo_0.5_Ir_0.5_O_3_ as examples, and evaluated the relationship between their structures and properties (Figure 11h). Moreover, the occurrence and order of leaching of Sr (A‐site) and Sc/Co (B‐site) can be controlled by adjusting the thermodynamic stability of the B‐site. Leaching at the A‐site increases the electrochemical surface area, while additional leaching at the B‐site generates a honeycomb IrO_x_H_y_ phase with increased intrinsic activity. SrCo_0.5_Ir_0.5_O_3_, which underwent surface reconstruction at bimetallic sites, exhibited the most excellent OER activity (Figure 11i). Grimaud et al.^[^ ^124^ ^]^ highlighted that the stability of Ir^(V)^‐based perovskite‐like catalysts was limited by the potential‐dependent dissolution/precipitation equilibrium of Ir species (Figure 11j).

#### 4.5.2 Surface Effect

Catalytic reactions are mainly dependent on the presence of active sites on the surface, and the surface structure of a catalyst has a great influence on its physical, chemical, and electronic structure.^[^ ^129^ ^]^ When the particle size of a material decreases, the ratio of the number of atoms on the surface to the total number of atoms increases, and the degree of coordination unsaturation increases, leading to a drastic change in catalytic properties.^[^ ^130^ ^]^ For example, the distribution of each type of atom at the surface sites (e.g., platforms, steps, edges, corners, etc.) of nanosheets, nanoneedles and nanoclusters, etc., varies with size. When the size decreases, the percentage of highly coordinated atoms (HCS) decreases continuously. In contrast, the number of low‐coordinated atoms (LCS) increases gradually at edges and corners. The different local geometries of the surface sites dramatically change the chemical bond breaking and catalytic properties.^[^ ^131^ ^]^ For example, 1D nanostructures can provide direct transport pathways and improved mass transfer, facilitating efficient electron transport. The ultrathin structure of 2D nanosheets can provide a large number of unsaturated ligand and dangling bonds, which can effectively stabilize reaction intermediates and lower the activation energy barrier.

**Figure 12:**
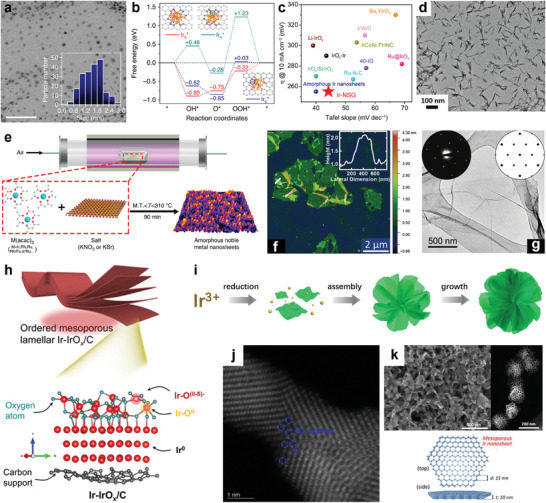
Other structure‐Surface Effect. a) Bright‐field TEM image (inset shows the corresponding particle size distribution of Ir nanoclusters based on a count of 200 in the sample area), scale bar: 20 nm. b) DFT calculation of the predicted free‐energy diagrams for OER at U = 1.23 V on Ir_S_^*^, Ir_N_^*^, and Ir_C_^*^ sites. c) Comparisons of overpotential (@10 mA cm^−2^) and Tafel slope for various state‐of‐the‐art noble metal‐based OER catalysts in acidic medium. Reproduced with permission.^[^ ^133^ ^]^ Copyright 2020, Springer Nature. d) TEM image of IrO_2_ NN‐L. Reproduced with permission.^[^ ^135^ ^]^ Copyright 2018, Wiley‐VCH. e) Schematic illustration of the general synthetic process for amorphous noble metal NSs. Note: M.T. is the melting point of metal acetylacetonate. Reproduced with permission.^[^ ^136^ ^]^ Copyright 2019, Springer Nature. f) AFM image and height profile taken along the white line of the exfoliated nanosheets. g) TEM image and (in inset) the experimental (left) and simulated (right) SAED patterns of a nanosheet along the [001] zone axis. Reproduced with permission.^[^ ^137^ ^]^ Copyright 2019, Wiley‐VCH. h) Illustration of the structure of the Ir‐IrO_x_/C‐20. Reproduced with permission.^[^ ^138^ ^]^ Copyright 2022, American Chemical Society. i) Schematic illustration on the growth mechanism of the 3D Ir superstructures. Reproduced with permission.^[^ ^139^ ^]^ Copyright 2016, American Chemical Society. j) Atomic STEM images of the RuO_2_ NSs. Reproduced with permission. Copyright 2020, Royal Society of Chemistry.^[^ ^140^ ^]^ k) TEM images and Scheme of mesoporous Ir nanosheet. Reproduced with permission.^[^ ^141^ ^]^ Copyright 2018, American Chemical Society.

However, due to the small particle size and high surface energy, ultrasmall nanoparticles are prone to agglomeration, which seriously affects the catalytic stability in practical applications. To overcome the obstacle, some researchers have proposed to immobilize the active sites Ir or Ru with non‐metallic elements to improve the catalytic stability. At the same time, the coordination environment of Ir or Ru can be changed to regulate the adsorption energy of reaction intermediates, thus improving the intrinsic catalytic activity.^[^ ^132^ ^]^ Liu et al.^[^ ^133^ ^]^ proposed a strategy for embedding ultrafine Ir nanoclusters into N,S‐doped graphene support (Ir‐NSG) (**Figure** **12**a). Unique electronic states and coordination environments for Ir site binding to N and S induce optimal oxygen intermediate binding energy and accelerate OER kinetics (Figure 12b,c). The ultrasmall Ru‐based catalysts were similarly modified by a similar strategy,^[^ ^134^ ^]^ such as encapsulation of trace amounts of Ru nanoclusters (NCs) into N‐vacancy (V_N_)‐rich g‐C_3_N_4_. Ru NCs/V_N_‐C_3_N_4_ has a unique porous structure and abundant defects, maximally exposing the active sites with ultra‐high mass activity.^[^ ^134b^ ^]^

Ultra‐thin nanocatalysts can expose a large number of unsaturated coordination sites, which can obtain significantly enhanced catalytic performance.^[^ ^142^ ^]^ Lee et al.^[^ ^135^ ^]^ reported ultrathin IrO_2_ nanoneedles (NN‐L) with diameters of ≈2 nm (Figure 12d). The enhanced metal conductivity leads to excellent OER activity of the nanoneedles. What's more, the ultrathin nanoneedle structure has a larger specific surface area, and the intrinsic and mass activities are enhanced. Li and co‐authors^[^ ^136^ ^]^ presented several amorphous noble metal nanosheets with thickness less than 10 nm (Figure 12e), which enable the modulation of the atomic arrangement of precious metals. The amorphous structure has abundant active sites and unique atomic structure, and the dynamic change of the valence state of Ir during the OER process gives it excellent catalytic performance. Notably, the nanosheet edges are the source of high OER activity, and the improved performance is attributed to the unsaturated coordination sites in the edges. The exfoliated hexagonal ruthenate nanosheets (Figure 12f,g) showed no significant change in the oxidation state and structure of the nanosheets during catalysis, with high activity and good stability at minimal mass loading.^[^ ^137^ ^]^

The active sites need to have a certain form of spatial distribution on the catalyst surface in order to be compatible with the reaction being catalyzed, i.e., the geometric effect. Zhao et al.^[^ ^138^ ^]^ proposed a nanoconfined self‐assembly strategy to design and prepare composite structures with Ir‐IrO_x_ nanoparticles uniformly distributed within Ir‐IrO_x_/C nanosheets (Figure 12h). Importantly, benefiting from the mixed valence, the abundance of electrophilic oxygen species (O^(II‐δ)−^), and the favorable mesoscopic structure, Ir‐IrO_x_/C reached 10 mA cm^−2^_geo_ at only 198 mV for acidic OER. Alternatively, ultrathin Ir nanosheets can be used as subunits to form 3D Ir superstructures (Figure 12i).^[^ ^139^ ^]^ Such interesting superstructure not only maximizes the exposure of active sites, but also has appropriate layer spacing as well as 3D accessible positions, which facilitates improved electrochemical energy conversion. Furthermore, defects are formed on the surface of ultrathin nanosheets, generating active sites with unsaturated coordination. For example, the presence of Ru vacancy defects on the surface of ultrathin RuO_2_ nanosheets significantly weakened the binding energy of O^*^ with respect to OOH^*^ and reduced the energy consumption for the conversion of O^*^ to OOH^*^, resulting in a significant enhancement of OER performance (Figure 12j).^[^ ^140^ ^]^ Much more interesting, the introduction of mesopores to ultrathin 2D materials can increase the number of unsaturated coordination sites such as edges, corners, etc., which will improve the utilization of the material and the availability of active sites. Yamauchi et al.^[^ ^141^ ^]^ designed a 2D mesoporous metallic Ir nanosheet (Figure 12k). Benefited from the abundance of catalytically active sites and the maximization of exposed surface atoms, it demonstrated excellent OER activity in acid.

#### 4.5.3 Facet‐Regulating

Benefiting from anisotropy, nanocatalysts with multiple crystalline facets usually exhibit facet‐dependent physical and chemical properties (e.g., geometrical structure, surface electronic structure, and redox active sites, etc.), which lead to differences in adsorption energies of OER intermediates, and thus exhibit different electrocatalytic activities.^[^ ^144^ ^]^ In general, the sides/edges of the catalysts are considered as active sites because they correspond to high index crystal faces with atomic arrangements and coordination unsaturation sites that are usually more favorable for the electrocatalytic reactions. Highly catalytically active crystalline surfaces can be exposed on the catalyst surface by the crystal surface modulation strategy. The exposed high‐activity facets can increase the mass specific activity of the active sites, lower the reaction energy barrier, and increase the catalytic reaction rate.^[^ ^145^ ^]^ Therefore, changes in the coordination environment of the active site can be driven by adjustments to the atomic arrangement, thereby altering the electron distribution state and achieving improved catalyst performance.

**Figure 13:**
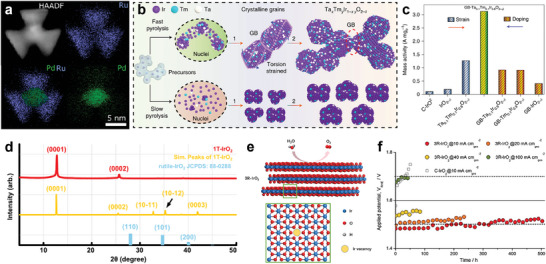
Crystal plane regulation of noble metal electrocatalysts. a) HAADF‐STEM image of a tripod nanoparticle and corresponding STEM‐EDX mapping showing Ru (blue) branches and Pd (green) core. Reproduced with permission.^[^ ^45c^ ^]^ Copyright 2018, American Chemical Society. b) The schematic routes for synthesizing GB‐Ta_0.1_Tm_0.1_Ir_0.8_O_2‐δ_ nanocatalyst via fast pyrolysis (top) versus nanoparticles without GB via slow pyrolysis (bottom). c) Mass activities of these nanocatalysts at *η* = 266 mV, showing the effects of both strain and doping on enhancing OER activities. Reproduced with permission.^[^ ^45d^ ^]^ Copyright 2021, Springer Nature. d) Comparison of XRD patterns of 1T‐IrO_2_ (red curve), simulation X‐ray diffraction peak (yellow curve) of 1T‐IrO_2_ and the rutile‐IrO_2_ (blue line) (JCPDS No. 88–0288). The XRD measurement showing high orientation of the layered structure of 1T‐IrO_2_ along the c‐axis. Reproduced with permission.^[^ ^47^ ^]^ Copyright 2021, Springer Nature. e) The structure scheme of 3R‐IrO_2_. f) Stability of 3R‐IrO_2_ by the chronopotentiometry technique at the constant current densities of 10, 20, 40, and 100 mA cm_geo_^−1^ and C‐IrO_2_ at the constant current density of 10 mA cm_geo_^−1^. Reproduced with permission.^[^ ^143^ ^]^ Copyright 2021, Elsevier Ltd.

Branched metal nanoparticles with highly defined features are benefit for catalysis, as the branches extend outward to give a high surface exposure, resulting in high overall activity.^[^ ^146^ ^]^ Tilley et al.^[^ ^45c^ ^]^ synthesized bimetallic branched and faceted Pd‐Ru nanoparticles with *fcc*‐Pd cores and *hcp*‐Ru branches with low index (10‐11) and facets (**Figure** **13**a). The branching and multifaceted structure improves the OER activity, and the low index crystal faces provide high stability, thus enabling higher stability to be obtained without sacrificing high activity. Jin and co‐authors^[^ ^45d^ ^]^ presented a torsion‐strained Ta_0.1_Tm_0.1_Ir_0.8_O_2‐δ_ nanocatalyst with numerous grain boundaries (Figure 13b), exhibiting a low overpotential of 198 mV to 10 mA cm^−2^. The synergistic interaction between the grain boundaries leads to the torsional strain of the Ir‐O bond and the doping‐induced ligand effect, which together modulate the adsorption energy of the oxygen intermediates, resulting in an improved catalytic activity while maintaining a good catalyst stability (Figure 13c).

Up to now, IrO_2_ is considered to be the most stable OER catalyst in acidic electrolytes. However, the rutile IrO_2_ catalysts that have been reported have slow OER kinetics and lack high intrinsic activity. The energy barrier for the rate‐determination step can be lowered by reducing the number of Ir‐O coordination sites, which in turn accelerates OER.^[^ ^147^ ^]^ Recent studies have revealed that the construction of sub‐stable nanostructures was expected to achieve excellent catalytic performance.^[^ ^148^ ^]^ Shao et al.^[^ ^47^, ^143^ ^]^ worked on the improvement of catalytic performance by designing metastable phase. With the combination effect of the optimized Ir reaction site, high number of surface unsaturated active sites in ultrathin 2D structure, and atomic utilization, the active site of Ir in 1T‐phase‐IrO_2_ provides an optimal free energy uphill in ^*^OH formation, leading to the high‐performance in acidic OER catalysis (Figure 13d).^[^ ^47^ ^]^ Another new structure 3R phase IrO_2_ (3R‐IrO_2_) (Figure 13e), in which the new active site of the edge‐shared structure and the rapid proton transport through the iridium vacancies along the inter‐ and intra‐layers.^[^ ^143^ ^]^ When used as an OER catalyst, an ultra‐low overpotential of only 188 mV was required to achieve a current density of 10 mA cm^−^2_geo_, while running smoothly for 511 h without significant decay.

#### 4.5.4 Doping and Supports

Heteroatom doping is an effective method to enhance the OER activity of Ru/Ir‐based electrocatalysts,^[^ ^149^ ^]^ and the enhancement of the activity originates from the introduction of unsaturated heterometallic atoms changing the environment around the heterogeneous metal atoms and causing a chain effect of the surrounding atoms.^[^ ^150^ ^]^ The heterogeneous metal atoms can interact with the surrounding atoms and subtly regulate the coordination state of the heterogeneous metal atoms, thus achieving the optimization and adjustment of the coordination environment.^[^ ^151^ ^]^ In addition, the valence states of heterogeneous metal atoms can be effectively tuned by metal vacancies, which is expected to achieve multivalent states.^[^ ^152^ ^]^

It is noteworthy that in the current studies targeting acidic OER catalysts, the stability issue is usually not given enough attention compared to the catalytic activity.^[^ ^153^ ^]^ In the OER process, the stability of catalysts is mainly determined by both mechanical and chemical factors. Among them, the mechanical stability is affected by the strength of the catalyst bonding to the supports/electrode and the stress of generating O_2_ bubbles. Chemical stability refers to the ability of catalyst active sites and supports to resist corrosion and oxidation. Currently, commonly used carbon supports are prone to corrosion at OER potentials, resulting in interfacial instability, active site loss, and performance degradation.^[^ ^154^ ^]^ The supports in loaded metal catalysts not only play the role of dispersing and stabilizing the metal nanoparticles, but also have strong interactions with the metal particles, which in turn affect the activity and stability of the catalysts.^[^ ^36^, ^155^ ^]^ Therefore, increasing the oxidation potential of supports by introducing O vacancies, regulating crystallinity, and doping with heteroatoms is crucial for the development of stable catalyst support materials.^[^ ^156^ ^]^

**Figure 14:**
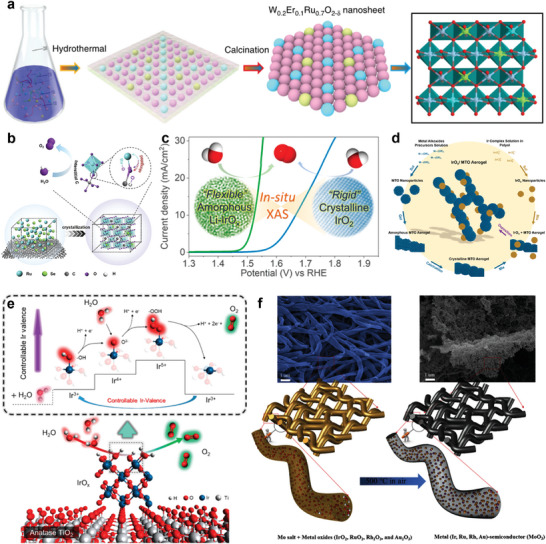
Construction of under‐coordinated active sites: doping and supporting. a) Schematic route for synthesis of W_0.2_Er_0.1_Ru_0.7_O_2−δ_ nanosheets. Reproduced with permission.^[^ ^157^ ^]^ Copyright 2020, Springer Nature. b) Schematic illustration of C‐RuO_2_‐RuSe‐10. Reproduced with permission.^[^ ^158^ ^]^ Copyright 2022, Elsevier Ltd. c) Schematic illustration of breaking long‐range order in Iridium oxide by alkali ion for efficient water oxidation. Reproduced with permission.^[^ ^50^ ^]^ Copyright 2019, American Chemical Society. d) Scheme of the synthesis of IrO_x_ nanoparticles supported on doped SnO_2_ aerogels. Reproduced with permission.^[^ ^159^ ^]^ Copyright 2020, American Chemical Society. e) Proposed OER mechanism of the ITOT catalyst. Reproduced with permission.^[^ ^160^ ^]^ Copyright 2019, American Chemical Society. f) SEM image of Mo salt, various metal oxides, PVP fibers (left), and metal‐metal oxide nanocomposites (right). Reproduced with permission.^[^ ^161^ ^]^ Copyright 2021, Springer Nature.

Ruthenium‐based catalysts follow the lattice oxygen oxidation mechanism, in which lattice oxygen participates in the reaction to generate oxygen vacancies (V_O_),^[^ ^44^, ^162^ ^]^ and Ru atoms exposed to the catalyst surface undergo oxidation to generate soluble high‐valent Ru^x>4^.^[^ ^35^ ^]^ Excessive oxidation of Ru inevitably leads to the collapse of the crystal structure and the destruction of stability.^[^ ^162^, ^163^ ^]^ Hence, Zhang et al.^[^ ^157^ ^]^ utilized W and Er co‐doping into the lattice of RuO_2_ to modify its electronic structure and avoid over‐oxidation of Ru (**Figure** **14**a). The doping of W and Er modulated the electronic structure of RuO_2_ through charge redistribution. The adsorption energy of oxygen intermediates was reduced while the oxygen vacancy generation energy was increased, thus inhibiting the formation of soluble Ru^x>4^, resulting in W_0.2_Er_0.1_Ru_0.7_O_2‐δ_ achieving a super‐low overpotential of 168 mV (10 mA cm^−2^) accompanied with a record stability of 500 h. A new strategy to stabilize RuO_2_ by introducing interstitial carbon (C‐RuO_2_‐RuSe) was proposed (Figure 14b),^[^ ^158^ ^]^ with the Ru‐O bonds elongating due to the formation of interstitial C. The elongated Ru‐O bonds in RuO_2_ enhance its stability and reduce energy barriers for OER. Unlike heteroatom doping with Ru to improve stability,^[^ ^164^ ^]^ Liu et al.^[^ ^50^ ^]^ obtained amorphous iridium oxide (Li‐IrO_x_) by incorporating of lithium ion into IrO_x_, showing outstanding OER activity (Figure 14c). Due to the higher oxidation state in Li‐IrO_x_, and the more flexible disordered [IrO_6_] octahedra are able to act as more electrophilic center, which effectively promotes the rapid reaction of the OER.

Support is also an important factor affecting the stability of OER catalysts, for which various supports have been investigated, such as nitrogen doped graphene,^[^ ^165^ ^]^ graphdiyne,^[^ ^166^ ^]^ TiN,^[^ ^167^ ^]^ and so on.^[^ ^168^ ^]^ Maillard et al.^[^ ^159^ ^]^ synthesized IrO_x_ NPs loaded on the doped SnO_2_ aerogels (IrO_x_/doped SnO_2_) and proved that the long‐term OER activity was related to the corrosion resistance of the dopant element and the concentration of the dopant element in the SnO_2_ matrix (Figure 14d). A composite catalyst IrO_x_‐TiO_2_‐Ti (ITOT) was reported to improve OER performance,^[^ ^160^ ^]^ and the high activity was derived from the controlled valence and high OH concentration in the catalysts (Figure 14e). Lee and co‐authors^[^ ^161^ ^]^ introduced electron‐deficient metal on semiconducting metal oxides‐consisting of Ir (Rh, Au, Ru)‐MoO_3_ embedded by graphitic carbon layers (IMO) to restructure highly electron‐deficient metal‐metal oxides (Figure 14f). The synergic effect of high oxidation state of MoO_3_ and the electron‐deficient Ir surface resist oxidative corrosion of the active site, thus exhibiting high stability and superior OER catalytic performance.

**Figure 15:**
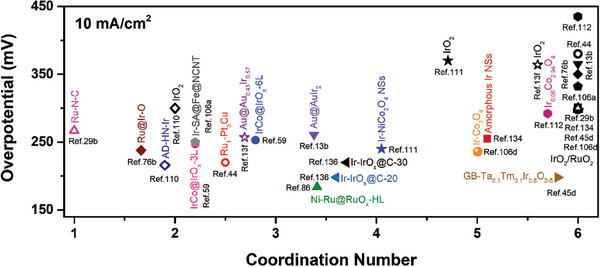
Structure‐activity relationships between the coordination number of active site and performance for the reported electrocatalysts.

To demonstrate more intuitively the relationship between the coordination Number and the performance, we selected representative cases for analysis, as shown in the **Figure** **15**, the catalysts with unsaturated coordination sites exhibit more excellent catalytic performance. At the same time, we should also point out that the overpotential to 10 mA cm^−2^ cannot be used as the only and main criterion for evaluating the electrocatalysts, because of the differences in catalyst loading, testing conditions, and electrode preparation methods in different studies. In order to make a more objective and comprehensive evaluation, we tabulated the performance parameters of the electrocatalysts mentioned in this review, as shown in **Tables** 1, 2, 3.

**Table 1:** Summary of the OER performance for the reported Ru‐based electrocatalysts in acidic electrolytes.

Catalysts	Electrolyte	Mass loading [*µ*g cm^−2^]	*η* [mV] to 10 mA cm^−2^	Mass activity [A mg^−1^]	Stability	Ref.
Ru@V‐RuO_2_/C HMS	0.5 m H_2_SO_4_	280.0	176	–	15 h @ 10 mA cm^−2^	[77]
Ni‐Ru@RuO_x_‐HL	0.5 m H_2_SO_4_	283.0	184	0.315 @ 1.45 V (Ru)	30 h @ 10 mA cm^−2^	[86]
Ru_1_‐PtCu_3_	0.1 m HClO_4_	16.3 (Pt+Ru)	220	6.615 @ 1.51 V (Ru)	28 h @ 10 mA cm^−2^	[44]
CaCu_3_Ru_4_O_12_	0.5 m H_2_SO_4_	250.0	171	1.942 @ 1.50 V (Ru)	24 h @ 10 mA cm^−2^	[106b]
Ru‐N‐C	0.5 m H_2_SO_4_	280.0	267	3.571 @ 1.497 V (metal)	30 h @ 1.49 V	[29b]
Ru_2_‐UiO‐67‐bpydc	0.5 m H_2_SO_4_	183.7	200	–	115 h @ 10 mA cm^−2^	[106f]
Ru(anc)−Co_3_O_4_‐5	0.5 m H_2_SO_4_	–	198.5	4.012 @ 1.50 V (Ru)	150 h @ 10 mA cm^−2^	[115]
(Ru, Mn)_2_O_3_	0.5 m H_2_SO_4_	849.2	168	–	40 h @ 10 mA cm^−2^	[117a]
Y_1.8_M_0.2_Ru_2_O_7−δ_	1 N H_2_SO_4_	12.5	≈350	≈0.2 @ 1.55 V	6 h @ 1 mA cm^−2^	[118]
YZRO/AB	0.5 m H_2_SO_4_	204.1	291	–	8 h @ 1 mA cm^−2^	[125e]
RuO_2_ Nanosheet	0.1 m HClO_4_	200.0	255	0.01 @ 1.455 V (Ru)	6 h @ 10 mA cm^−2^	[137]
RuO_2_ NSs	0.5 m H_2_SO_4_	125.0	199	0.52 @ 1.46 V (Ru)	6 h @ 10 mA cm^−2^	[140]
Pd‐Ru branched NPs	0.1 m HClO_4_	408.2	225	–	120 min @ 10 mA cm^−2^	[45c]
Au‐Ru branched NPs	0.1 m HClO_4_	408.2	220	–	200 CV cycles between 1 and 1.53 V	[146b]
Co_0.11_Ru_0.89_O_2‐δ_	0.5 m H_2_SO_4_	283.1	169	–	50 h @ 10 mA cm^−2^	[149c]
Nb_0.1_Ru_0.9_O_2_	0.5 m H_2_SO_4_	510	204	–	360 h @ 200 mA cm^−2^	[149d]
Mo_0.15_‐RuO_2_	0.5 m H_2_SO_4_	≈230	147	0.6637 @ 1.48 V	20 h @ 10 mA cm^−2^	[150b]
RuCoO_x_@LLCF,	0.1 m HClO_4_	10.0 (Ru)	256 ± 5	1.9114 @ 1.48 V (Ru)	110 h @ 1.51 V	[151b]
BCN‐0.5Ru	0.5 m H_2_SO_4_	102.0	164	–	12 h @ 10 mA cm^−2^	[156a]
Ru/S NSs400	0.5 m H_2_SO_4_	10.0 (Ru)	219	0.613	600 h @ 10 mA cm^−2^	[156d]
Re_0.06_Ru_0.94_O_2_	0.1 m HClO_4_	100	190	0.5 @ 1.502 V 7.811 @1.502 V (Re+Ru)	200 h @ 10 mA cm^−2^	[152]
W_0.2_Er_0.1_Ru_0.7_O_2‐δ_	0.5 m H_2_SO_4_	330.0	168	1.519 @ 1.505 V (ox)	500 h @ 10 mA cm^−2^	[157]
C‐RuO_2_‐RuSe‐5	0.5 m H_2_SO_4_	60.0 (Ru)	212	–	50 h @ 50 mA cm^−2^	[158]
75‐H‐RuO_2_	0.5 m H_2_SO_4_	255.1	200	–	20 h @ 10 mA cm^−2^	[162c]
Li_0.52_RuO_2_	0.5 m H_2_SO_4_	637 (Ru)	156	–	70 h @ 10 mA cm^−2^	[164a]
UfD‐RuO_2_/CC	0.5 m H_2_SO_4_	520	179	–	20 h @ 10 mA cm^−2^	[168a]
RuO_2_/(Co,Mn)_3_O_4_	0.5 m H_2_SO_4_	–	270	0.3665 @ 1.53 V (Ru)	24 h @ 10 mA cm^−2^	[168c]
H/d‐MnO_x_/RuO_2_	0.5 m H_2_SO_4_	–	178	≈0.325 @ 1.45 V	40 h @ 10 mA cm^−2^	[168d]

**Table 2:** Summary of the OER performance for the reported Ir‐based electrocatalysts in acidic electrolytes.

Catalysts	Electrolyte	Mass loading [*µ*g cm^−2^]	*η* [mV] to 10 mA cm^−2^	Mass activity [A mg^−1^]	Stability	Ref.
IrW NDs	0.1 m HClO_4_	10.2	300 @ 8.1 mA cm^−2^	0.794 @ 1.53 V (Ir)	3000 CV cycles between 1.25 and 1.7 V	[60]
Rh_22_Ir_78_ NPs	0.5 m H_2_SO_4_	280.0	292	1.17 @ 1.53 V (Ir)	2000 CV cycles between 1.1 and 1.5 V_Ag/AgCl_	[68]
Pt_62_Co_23_/Ir_15_ FBNWs	0.1 m HClO_4_	1.98 (Ir)	308	–	10 h @ 1.50 V	[71]
W‐Ir‐B	0.5 m H_2_SO_4_	78.9 (Ir)	291	0.518 @ 1.53 V (Ir)	120 h @ 100 mA cm^−2^	[13a]
IrCuNi DCNCs	0.1 m HClO_4_	6.0 (Ir)	273	6.6 @ 1.53 V (Ir)	1800 CV cycles between 1.2 and 1.7 V	[74]
IrFeCoNiCu‐HEA	0.1 m HClO_4_	288.0 (Ir)	302	0.03467 @ 1.53 V (Ir)	12 h @ 10 mA cm^−2^	[75]
IrGa‐IMC@IrO_x_	0.1 m HClO_4_	20.0 (Ir)	272	0.841 @ 1.52 V (Ir)	3000 CV cycles between 0.8 to 1.6 V	[79]
Ir_6_Ag_9_ NTs	0.5 m H_2_SO_4_	13.3 (Ir)	285	–	6 h @ 5 mA cm^−2^	[80]
α‐Ni(OH)_2_@Ir	0.5 m H_2_SO_4_	285.0	238	2.74 @ 1.55 V (Ir)	1000 CV cycles between 1.2 to 1.6 V	[82]
Ir/Fe_4_N	0.5 m H_2_SO_4_	76.5 (Ir)	316 ± 5	0.1164 @ 1.54 V (Ir)	120 h @ 10 mA cm^−2^	[83]
Ir_3_Cu	0.1 m HClO_4_	25.5 (Ir)	298	–	5000 potential cycles test	[84]
Pt_0.1_La_0.1_‐IrO_2_@NC	0.5 m H_2_SO_4_	550.0	205	–	135 h @ 10 mA cm^−2^	[87]
Pd@Ir_3L_/C	0.1 m HClO_4_	10.2 (Ir)	263	3.330 @ 1.53 V (Ir)	2000 CV cycles between 1.3 to 1.6 V	[88]
IrNiO_x_	0.05 m H_2_SO_4_	10.2 (Ir)	–	0.676 @ 1.53 V (Ir)	–	[90]
Pd@Ir c‐nc	0.1 m HClO_4_	5.4 (Ir)	–	1.079 @ 1.53 V (Ir)	10 h @ 1.53 V	[91]
Au@AuIr_2_	0.5 m H_2_SO_4_	20.0 (Ir)	261	1.44 @ 1.53 V (Ir)	40 h @ 10 mA cm^−2^	[13b]
H‐*γ*‐SIO‐3	0.1 m HClO_4_	210.0	200	0.466 @ 1.50 V (Ir)	1080 h @ 10 mA cm^−2^	[96]
IrNiCu DNF/C	0.1 m HClO_4_	20.0 (Ir)	302 ± 7	0.46 ± 0.07 @ 1.53 V (Ir)	2500 CV cycles between 1.2 to 1.7 V	[98]
Ir_44_Pd_10_	0.1 m HClO_4_	12.5 (Ir)	226	1.99 @ 1.48 V (Ir)	10 000 CV cycles between 1.2 to 1.6 V	[13c]
Cu_1.11_Ir	0.05 m H_2_SO_4_	142.9	286	0.073 @ 1.51 V (Ir)	5 h @ 1 mA cm^−2^	[100a]
Pt_39_Ir_10_Pd_11_	0.1 m HClO_4_	8.4 (Pt+Ir+Pd)	408	0.20 @ 1.53 V (Pt+Ir)	–	[101]
Ir−NiCo_2_O_4_ NSs	0.5 m H_2_SO_4_	1000.0	240	10.0 @ 1.467 V (Ir)	70 h @ 10 mA cm^−2^	[108]
Ir_0.06_Co_2.94_O_4_	0.1 m HClO_4_	5.0 (Ir)	292	2.511 @ 1.53 V (Ir)	200 h @ 10 mA cm^−2^	[109]
Ir‐SA@Fe@NCNT	0.5 m H_2_SO_4_	1.14 (Ir)	250	13.7 @ 1.50 V (Ir)	12 h @ 1.48 V	[106a]
SrTi(Ir)O_3_	0.1 m HClO_4_	280.0	265	≈0.525 @ 1.53 V (Ir)	20 h @ 10 mA cm^−2^	[45a]
Ni_0.34_Co_0.46_Ir_0.2_O_δ_	0.1 m HClO_4_	200.0	280	–	40 000 s @ 10 mA cm^−2^	[106c]
Ir‐Co_3_O_4_	0.5 m H_2_SO_4_	255.0	236	3.34337 @ 1.53 V (Ir)	30 h @ 10 mA cm^−2^	[106d]
Ir‐STO	0.1 m HClO_4_	210.0	247	0.82 @ 1.525 V (Ir)	20 h @ 10 mA cm^−2^	[106e]
h‐HL‐Ir SACs	0.1 m HClO_4_	500.0	216	1.202 @ 1.489 V (Ir)	60 h @ 10 mA cm^−2^	[106g]
Ir_0.1_Ta_0.9_O_2.45_	0.1 m HClO_4_	4.8 × 10^12^ Ir atoms	–	1.2 ± 0.5 @ 1.55 V (Ir)	24 h @ 1.60 V	[114]
Ir–MnO_2_	0.5 m H_2_SO_4_	1000.0	218	0.766 @ 1.53 V (Ir)	650 h @ 10 mA cm^−2^	[116]
6H‐SrIrO_3_	0.5 m H_2_SO_4_	≈900	248	–	30 h @ 10 mA cm^−2^	[120]
IrO_x_/SrIrO_3_	0.5 m H_2_SO_4_	–	270	–	30 h @ 10 mA cm^−2^	[122]
Sr_2_CoIr^(V)^O_6_	0.1 m HClO_4_	50.0	330	–	24 h @ 10 mA cm^−2^	[124]
Bi_2_Ir_2_O_7_	0.1 m HClO_4_	102.0	–	0.034 @ 1.525 V	500 CV cycles between 1.0 to 1.6 V	[125a]
IrO_x_/9R‐BaIrO_3_	0.5 m H_2_SO_4_	283.0	230	0.168 @ 1.46 V (Ir)	48 h @ 10 mA cm^−2^	[126a]
SrIr_2_O_6_	0.1 m HClO_4_	280.0	303	0.0613 (Ir)	300 h @ 10 mA cm^−2^	[126b]
Ir‐NSG	0.1 m HClO_4_	300.0	265	1.21162 @ 1.53 V (metal)	–	[133]
IrO_2_ NN‐L	1 m H_2_SO_4_	250 (oxide)	313	≈0.05 @ 1.55 V (oxide)	–	[135]
Ir NSs	0.1 m HClO_4_	204.1	255	0.2218 @ 1.53 V (Ir)	8 h @ 1.485 V	[136]
Ir‐IrO_x_/C‐20	0.5 m H_2_SO_4_	204.0	198	–	18 h @ 1.45 V	[138]
3D Ir superstructures	0.1 m HClO_4_	11.5 (Ir)	270	–	8 h @ 2.5 mA cm^−2^	[139]
	0.5 m H_2_SO_4_	11.5 (Ir)	250	–	8 h @ 2.5 mA cm^−2^	
mesoporous Ir NSs	0.5 m H_2_SO_4_	135.8	240	0.26 @ 1.50 V (Ir)	8 h @ 10 mA cm^−2^	[141]
IrO_2_@Co_3_O_4_‐CoMoO_4_	0.5 m H_2_SO_4_	459.0	236	0.713 @ 1.53 V (Ir)	36 h @ 10 mA cm^−2^	[132]
Ir WNWs	0.5 m HClO_4_	31.0 (Ir)	270	–	25 000 s @ 5 mA cm^−2^	[142a]
1T‐IrO_2_	0.1 m HClO_4_	≈200	197	–	45 h @ 50 mA cm^−2^	[47]
3R‐IrO_2_	0.1 m HClO_4_	255	188	0.691 @ 1.53 V (Ir)	511 h @ 10 mA cm^−2^	[143]
amorphous‐IrO_2_	0.5 m H_2_SO_4_	204	231	0.0586 @ 1.53 V	100 h @ 10 mA cm^−2^	[147]
IrO_2_NR	0.5 m H_2_SO_4_	–	205	2.3545 @ 1.50 V (Ir)	500 ks @ 10 mA cm^−2^	[148]
Li‐IrSe_2_	0.5 m H_2_SO_4_	250	220	0.06616 @ 1.45 V (Ir)	10 h @ 20 mA cm^−2^	[149a]
350‐Ta@IrO_x_	0.5 m H_2_SO_4_	≈200	223	1.2074 @ 1.55 V (Ir)	500 h @ 10 mA cm^−2^	[149e]
IrCoO_x_@LLCF	0.1 m HClO_4_	10.0 (Ir)	286 ± 5	1.0135 @ 1.48 V (Ir)	233 h @ 1.55 V	[151b]
CrO_2_‐0.16IrO_2_	0.5 m H_2_SO_4_	50.0 (Ir)	353 @ 200 mA cm^−2^	0.762 @ 1.53 V (Ir)	100 h @ 1000 mA cm^−2^	[155b]
Ir‐ZrTaO_x_	0.5 m H_2_SO_4_	22.5 (GCE)	254.07	1.0651 @ 1.53 V (Ir)	–	[156b]
IrO_x_‐TiO_2_‐Ti	0.05 m H_2_SO_4_	580 (IrO_x_)	200	–	100 h @ 10 mA cm^−2^	[160]
IrO_2_‐MoO_3_	0.5 m H_2_SO_4_	50.0 (GCE)	156	0.1789 @ 1.43 V (Ir)	50 h @ 10 mA cm^−2^	[161]
Ir@N‐G‐750	0.5 m H_2_SO_4_	23.0 (Ir)	303	2.42 @ 1.607 V	2000 potential cycles between 0.95 and 1.75 V	[165]
IrO_2_@Ir/TiN	0.5 m H_2_SO_4_	379	265	0.2223 @ 1.55 V (Ir)	6 h @ 10 mA cm^−2^	[167]
Li‐IrO_x_	0.5 m H_2_SO_4_	125.0	270	0.10 @ 1.52 V (Ir)	10 h @ 40 mA cm^−2^	[50]
40‐IG	0.5 m H_2_SO_4_	204.1	276	1.280 @ 1.6 V	4 h @ 20 mA cm^−2^	[169]
Au@Ir NRBs	0.5 m H_2_SO_4_	–	296	0.694 @ 1.55 V (Ir)	–	[170]

**Table 3:** Summary of the OER performance for the reported RuIr‐based electrocatalysts in acidic electrolytes.

Catalysts	Electrolyte	Mass loading [*µ*g cm^−2^]	*η* [mV] to 10 mA cm^−2^	Mass activity [A mg^−1^]	Stability	Ref.
Ru_1_Ir_1_Ox	0.5 m H_2_SO_4_	150.0	204	1.1246 @ 1.53 V (Ru+Ir)	110 h @ 100 mA cm^−2^_geo_	[69]
RuIr‐NC	0.05 m H_2_SO_4_	150.0	165	0.796 @ 1.45 V (Ru+Ir)	122 h @ 1 mA cm^−2^	[46a]
Ru_0.5_Ir_0.5_O_2_	0.5 m H_2_SO_4_	283.0	151	0.7304 @ 1.44 V (Ru+Ir)	618.3 h @ 100 mA cm^−2^	[72]
Ru@IrO_x_	0.05 m H_2_SO_4_	50.0	282	0.650 @ 1.55 V (IrO_x_)	24 h @ 1.55 V	[76a]
Ru@Ir–O	0.5 m H_2_SO_4_	350.0	238	1.169 @ 1.55 V (Ru+Ir)	40 h @ 10 mA cm^−2^	[76b]
IrRu@Te	0.5 m H_2_SO_4_	150.0	220	0.590 @ 1.50 V (Ru+Ir)	20 h @ 10 mA cm^−2^	[81]
RuIr@CoNC	0.5 m H_2_SO_4_	50.0 (Ru+Ir)	223	2.041 @ 1.53 V (Ru+Ir)	40 h @ 10 mA cm^−2^	[89]
RuIrO_x_	0.5 m H_2_SO_4_	10.0 (Ru+Ir)	233	–	3000 CV cycles between 1.0 to 1.5 V	[99]
Co‐RuIr alloy	0.1 m HClO_4_	–	235	–	25 h @ 10 mA cm^−2^	[171]

Although a wide variety of electrocatalysts have been reported based on the construction of under‐coordinated sites, which greatly improves the activity or stability of the catalysts. Increased activity is usually achieved at the expense of stability and vice versa, and it remains difficult to achieve excellent activity and stability at the same time. Currently, stability is usually not given enough attention in the study of acidic OER catalysts compared to activity. However, OER stability plays an indispensable role in commercial applications, and the activity‐stability trade‐off of highly active catalysts must be resolved in order to meet the demands of practical applications. In addition, due to the inherent high potential and strong corrosive environment of acidic OER, the catalysts undergo a reconfiguration process, and the composition and catalytic behavior of the catalysts are greatly altered. The exact nature of the active sites for these reactions remains a matter, such as all Ir^3+^, Ir^4+,^ and Ir^5+^ have been considered as active sites. Its exact structure cannot be judged even by in situ characterization since the amorphous structures are generated during the OER process and surface reconstruction may also dissolve instantaneously. Thus, more accurate in situ devices and computational simulations are needed to verify that. Moreover, catalyst development is typically time‐consuming and requires iterative testing at a high cost. Machine learning can be utilized to incorporate multiple parameters into a complex dynamic reaction process, screen the optimal catalyst, and finally experimentally validate it. Machine learning guides the design of long‐life and efficient OER catalysts to aid the development of acidic electrolyzed water.

## 5 PEMWEs

**Figure 16:**
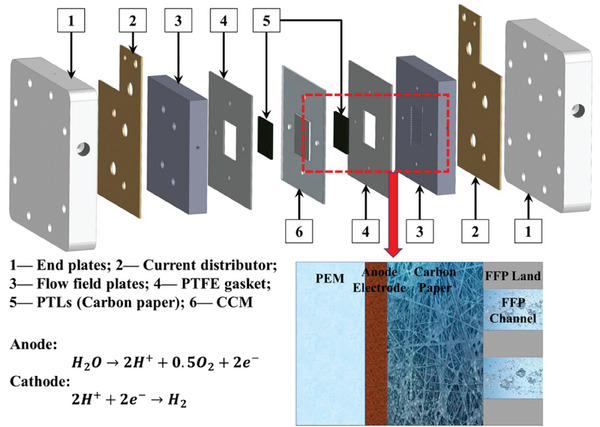
Schematic of typical PEMWE cells and components around anode electrode. Reproduced with permission.^[^ ^172^ ^]^ Copyright 2022, MDPI.

PEMWE is mainly composed of membrane electrodes consisting of proton exchange membranes, catalysts and gas diffusion layers, and bipolar plates. **Figure** **16** shows a schematic diagram of the principle and basic components of a PEMWE cell,^[^ ^172^ ^]^ with the membrane electrode assembly (MEA) as the core component, which is mainly composed of a proton exchange membrane, an anode catalyst layer, and a cathode catalyst layer.^[^ ^7^, ^173^ ^]^ The PEMWE uses a bipolar design that allows it to operate at high pressure differentials across the membrane. The process of PEM water electrolysis involves first supplying water to the anode via a pump, where the water is broken down into O_2_, protons (H^+^), and electrons (e^−^), which pass through a proton exchange membrane into the cathode.^[^ ^174^ ^]^ Electrons flow out of the anode and through the power supply circuit to the cathode while the power supply provides the driving force (cell voltage). On the cathode side, two protons and electrons recombine to produce H_2_. During the whole process, the anodic oxygen precipitation reaction produces a large amount of H^+^, resulting in a strongly acidic state of the anode, which requires high corrosion resistance of the materials used in the anode environment, and also needs to ensure a stable reaction at a certain voltage (≈2 V), making the study of the anode particularly important.^[^ ^7b^ ^]^

When the electrolytic cell is in operation, the electrolytic reaction occurs mainly at the solid‐liquid‐gas three‐phase interface, which consists of the electrocatalyst (the solid‐phase portion, which serves as a carrier for electron transport), the water and the Nafion membrane (the liquid‐phase portion, which provides and transfers protons in the anodic reaction), and the gas‐diffusion layer (the gas‐phase portion, which enables the formation of a gas channel inside the catalytic layer and provides gas transport).^[^ ^9^, ^175^ ^]^ During the actual reaction, the actual reaction voltage is greater than the theoretical voltage due to the presence of overpotentials. Three main factors are involved in the generation of overpotentials: activation overpotentials due to losses generated during electrochemical activation, ohmic overpotentials due to the material body resistance and the interface resistance between the different layers, and diffusion overpotentials due to the impeded transfer of reactants and products.^[^ ^6^, ^175^, ^176^ ^]^

Therefore, the optimization of electrode structure and preparation based on OER catalysts is crucial.^[^ ^7b^ ^]^ The role of the gas diffusion layer is to transport water and hydrogen or oxygen to the catalytic layer and to provide an electron transfer channel.^[^ ^177^ ^]^ Thus, the gas diffusion layer must have appropriate porosity and pore size, good electrical conductivity, and stability in order to fulfil the corresponding functions.^[^ ^178^ ^]^ The thickness of the catalytic layer of the currently prepared membrane electrode is 5–15 µm, and the thickness of the Nafion membrane is generally 25–150 µm.^[^ ^179^ ^]^ A Nafion membrane that is too thick will increase the internal resistance of the cell, while one that is too thin will reduce the lifetime of the membrane.^[^ ^179a^ ^]^ Müller et al. investigated the improvement of water diffusion by reducing the thickness of the catalyst coating film and found a significant correlation between film thickness and water transport.^[^ ^180^ ^]^ However, further reduction of the film thickness does not significantly improve the overall performance, which ultimately needs to be achieved by changing the catalyst.

The preparation of membrane electrodes for PEMWE has gone through two generations of development, namely the gas diffusion electrode (GDE) method and the catalyst coated membrane (CCM) method.^[^ ^181^ ^]^ In the GDE method, a gas diffusion layer (GDL) is used as the support layer, and the catalyst is covered on the surface of the diffusion layer. Then, the membrane electrode is obtained by hot‐pressing the Nafion membrane with the diffusion layer. This method is simple and easy to prepare, but the catalyst of the finished membrane electrode will be left in the gas diffusion layer, resulting in less contact area between the catalyst and the Nafion membrane, which reduces the utilization rate of the catalyst.^[^ ^182^ ^]^ The CCM method is the mainstream method in commercial use at present, which is to use the Nafion membrane as the support layer, and then the catalyst will be covered on the surface of the Nafion membrane through the methods of hot pressing, electrochemical deposition, spraying, etc.^[^ ^183^ ^]^ The gas diffusion layer is placed on both sides for hot pressing, and the catalyst will be pressed onto the surface of the membrane electrodes. The catalyst is covered on the surface of Nafion membrane by hot pressing, electrochemical deposition, spraying, etc., and then the gas diffusion layer is placed on both sides for hot pressing to form the CCM three‐in‐one membrane electrode. This method is currently the most widely used, and the resulting membrane electrode module has the advantage of low impedance due to the closer contact between the catalytic layer and the proton exchange membrane.^[^ ^184^ ^]^ However, the multiphase transport channels in the catalytic layer are in a disordered state, and there are strong electrochemical polarization and concentration polarization, which restrict the high‐current discharge performance of the membrane electrode.^[^ ^185^ ^]^

In the practical use of PEMWE, MEAs need to be operated at high current densities (≥1–2 A cm^−2^) to ensure efficient hydrogen production, and thus low catalyst utilization, high ohmic resistance, and limited mass transfer need to be addressed simultaneously.^[^ ^185^, ^186^ ^]^ Constructing ordered‐structure MEAs is expected to simultaneously reduce electrocatalytic kinetics, mass transfer, and ohmic losses, which is a pursued but challenging goal of PEMWE hydrogen production research. Among them, the development of low‐cost and high‐efficiency catalysts is the key to MEA research, and the construction of unsaturated catalytic sites not only improves the intrinsic activity of the catalysts, but also greatly reduces the high cost caused by excess metals. Therefore, the study of rational design and oriented construction of coordination unsaturated active sites provides low‐cost, high‐efficiency, and stable catalysts as well as new ideas and theoretical basis for the development of catalysts for the scale‐up of hydrogen production in PEMWE.

## 6 Conclusions and Perspectives

**Figure 17:**
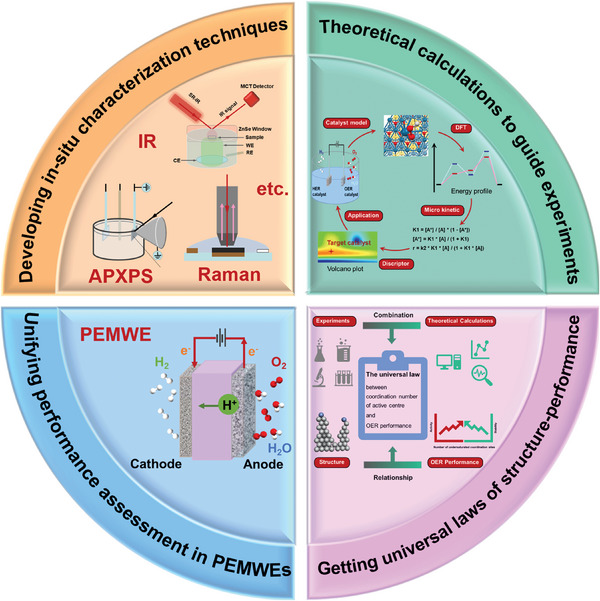
Prospects for the production of hydrogen energy from acidic water electrolysis.

With the development of characterization techniques, especially in situ characterization, which provides a powerful help in revealing the dynamic changes of catalysts and reaction mechanisms in acidic media, important results have been achieved in recent years in developing efficient and stable electrocatalysts. In this paper, the design of noble metal‐based OER electrocatalysts and the research progress in recent years are reviewed from the construction of unsaturated sites, with emphasis on the reaction and degradation mechanisms in acidic media. In addition, the research progress of PEMWEs is discussed at the device level. However, there is still a gap between the developed catalysts and practical industrial applications, and some challenges are still faced in critical areas. In order to accelerate the development of hydrogen production from electrolytic water, more in‐depth research is needed in the following areas (**Figure** **17**), such as developing in‐situ characterization techniques, theoretical calculations to guide experiments, getting universal laws of structure‐performance and unifying performance assessment in PEMWEs.

### 6.1 Development of In Situ Electrochemical Non‐Destructive Characterization Techniques

Clarifying the OER reaction and catalyst decay mechanism can guide the iterative upgrading of high‐performance catalysts. The in situ monitoring of the dynamic structural evolution of active sites and reactive species during electrocatalysis is a key link in resolving the catalyst conformational relationship. However, facing the problems of high fluid interference, weak signals, and fast dynamic evolution of catalyst surface under high voltage and strong corrosive medium, it increases the difficulty of identifying the true active phase and gaining a deeper understanding of the reaction mechanism. Therefore, there is an urgent need to develop high‐resolution, high‐sensitivity, and multi‐dimensional in‐situ characterization techniques for electrocatalytic surfaces under working conditions, such as differential electrochemical mass spectrometry (DEMS), Raman/infrared spectroscopy (IR), TEM, XAS, and near‐atmospheric‐pressure XPS. It is also hoped to propose methods to improve the temporal, spatial and energy resolution as well as to design in situ electrolytic cells suitable for PEM systems to carry out multi‐scale in situ studies. To study the dynamic evolution of electrocatalytic interfaces under working conditions, reaction intermediate states involved on the catalyst surface, surface dissolution corrosion and compositional changes, and activation/regulation mechanisms of lattice oxygen atoms.

### 6.2 Theoretical Calculations to Guide Experiments

Various descriptors obtained from theoretical calculations can be used to guide the development of efficient catalysts. The most common means currently used is to design catalysts in two steps: (1) finding descriptors that can describe the catalytic activity; and (2) changing the descriptors to help us design the catalyst. For the design of unsaturated sites of OER catalysts, the correlation between the atomic structure, chemical bonding, and local coordination of noble metal catalytic materials and their intrinsic catalytic activities can be revealed. Potential constitutive relationships between material properties and performance are explored through theoretical calculations, and multidimensional descriptors affecting catalyst performance are refined. Establish physically interpretable correlation models and develop high‐throughput screening strategies based on machine learning to identify breakthrough directions for synergistic enhancement of catalytic activity and stability.

### 6.3 Establishment of Constructive Relationships

Understanding the constitutive relationship of metal catalysts is one of the important means to study the reaction mechanism and construct the optimal catalytic system. Theoretical calculations are matched with experiments to establish the universal law between the coordination number of active center and OER performance. To establish catalytic models by combining the results of in situ characterization, to perceive the nature of catalyst activity and reaction mechanism at atomic and molecular levels. Comparison and correlation of factors such as specific chemical bond activation patterns and intermediate adsorption on the surface of different catalyst models will dramatically improve the research in catalysis and catalyst design/development at the molecular level.

### 6.4 Harmonization of Performance Assessment in PEMWEs

In most of the previous studies on acidic OER catalysts, tests of activity and stability have only been performed at small current densities. We call for performance testing of catalysts at higher current densities, especially in PEMWE devices, to meet the standards for industrial applications in the future. In addition, there is an urgent need to establish uniform activity and stability rating criteria to enable quantitative comparison of the performance of different electrocatalysts. Given the differences between three‐electrode and PEMWEs, the electrode structure (MEA), electric operating conditions such as potential, current density, temperature, air pressure, and flow field, and other factors such as bubble formation, desorption, diffusion and escape, and transfer processes in porous electrodes also need to be considered. In general, PEMWE for industrial applications requires long‐term operation more than 50 000 h. As well it should operate stably at 2.0 A cm^−2^ with an estimated cost of US$2 per kg of H_2_ in 2030, the target set by the US Department of Energy. Nevertheless, the expense still cannot compete with that for fossil‐fuel‐derived H_2_. Therefore, future goal will be to cost less than US$1 per kg of H_2_.

The large‐scale application of PEMWE depends on the development of acidic OER catalysts. The preparation of OER catalysts with high activity and long term stability for high current densities is still a great challenge. In this review, the characteristics of AEM and LOM are summarized from the reaction mechanism, and the relationship between unsaturated sites and activity is systematically elucidated from the viewpoint of coordination chemistry. Then we summarize the structures of four types of unsaturated coordination sites, including alloys, core‐shells, nanoframes, and single atoms, and propose strategies to improve the activity and stability of the catalysts. The structure‐activity relationship of catalysts is precisely analyzed from the point of view of the construction of surface unsaturated coordination catalytic active sites, and identifies catalyst active sites at the atomic level and understands the molecular reaction mechanisms occurring at the active sites, which provides a rational and predictable approach for the design of future acidic OER catalysts. Finally, it is hoped that the study in this paper will provide some suggestions and guidance for the design and development of efficient and long‐lived OER catalysts in the future. We firmly believe that through the joint efforts of material design, advanced characterization techniques, theoretical modeling, and device development, which can help the industrialization of PEMWE technology and build a clean, low‐carbon, safe, and efficient modern energy system.

## Conflict of Interest

The authors declare no conflict of interest.
